# Temperature Effects During a Sublethal Chronic Metal Mixture Exposure on Common Carp (*Cyprinus carpio*)

**DOI:** 10.3389/fphys.2021.651584

**Published:** 2021-03-16

**Authors:** Giovanni Castaldo, Marion Pillet, Leen Ameryckx, Lieven Bervoets, Raewyn M. Town, Ronny Blust, Gudrun De Boeck

**Affiliations:** Systemic Physiological and Ecotoxicological Research (SPHERE), Department of Biology, University of Antwerp, Antwerp, Belgium

**Keywords:** *Cyprinus carpio*, temperature, metal pollution, ion-homeostasis, ionoregulation, mixture stress

## Abstract

The aquatic environment is the final sink of various pollutants including metals, which can pose a threat for aquatic organisms. Waterborne metal mixture toxicity might be influenced by environmental parameters such as the temperature. In the present study, common carp were exposed for 27 days to a ternary metal mixture of Cu, Zn, and Cd at two different temperatures, 10 and 20°C. The exposure concentrations represent 10% of the 96 h-LC_50_ (concentration lethal for the 50% of the population in 96 h) for each metal (nominal metal concentrations of Cu: 0.08 μM; Cd: 0.02 μM and Zn: 3 μM). Metal bioaccumulation and toxicity as well as changes in the gene expression of enzymes responsible for ionoregulation and induction of defensive responses were investigated. Furthermore the hepatosomatic index and condition factor were measured as crude indication of overall health and energy reserves. The obtained results showed a rapid Cu and Cd increase in the gills at both temperatures. Cadmium accumulation was higher at 20°C compared to 10°C, whereas Cu and Zn accumulation was not, suggesting that at 20°C, fish had more efficient depuration processes for Cu and Zn. Electrolyte (Ca, Mg, Na, and K) levels were analyzed in different tissues (gills, liver, brain, muscle) and in the remaining carcasses. However, no major electrolyte losses were observed. The toxic effect of the trace metal ion mixture on major ion uptake mechanisms may have been compensated by ion uptake from the food. Finally, the metal exposure triggered the upregulation of the metallothionein gene in the gills as defensive response for the organism. These results, show the ability of common carp to cope with these metal levels, at least under the condition used in this experiment.

## Introduction

Metal pollution, as a result of rapid industrialization and urbanization, is increasing in fresh water systems. Anthropogenic inputs, through mining activities, industries, and urbanization play a major role in the release of metals in the aquatic environment (Sevcikova et al., 2011). Since metal ions can be easily bioaccumulated, and are non-biodegradable and potentially toxic to the aquatic biota, they are of great concern (Feng et al., 2015). Among metals, copper (Cu^+^), zinc (Zn^2+^), and cadmium (Cd^2+^) are perhaps some of the most studied pollutants due to their ecological importance and adverse effects on the organisms. Copper (Cu^+^) and zinc (Zn^2+^) are considered essential trace metals and are necessary for many metabolic processes, but they can be toxic when present in excess (Bury et al., 2003; Strižak et al., 2014). Cadmium (Cd^2+^) on the other hand is considered a non-essential element (McGeer et al., 2011). Due to their harmful effects on aquatic life, these metals are categorized as priority pollutants in many countries in the world (Grosell, 2011; Hogstrand, 2011; McGeer et al., 2011; Strižak et al., 2014).

Metal ions can be accumulated by organisms living in polluted areas via direct uptake through the gills or via food (Perera et al., 2015). Copper, Zn, and Cd can be taken up via gills through several ion channels (Niyogi and Wood, 2003). Copper can be taken up via the high-affinity copper transporter (CTR1), the divalent metal transporter (DMT1) (Mackenzie et al., 2004; Grosell, 2011; Komjarova and Bury, 2014) or via a putative Na^+^-channel, coupled with H^+^-ATPase (Grosell, 2011). Copper can impact Na^+^ homeostasis and interfere with ionoregulation affecting the activity of the Na^+^/K^+^-adenosine triphosphate (Na^+^/K^+^-ATPase) (Wilson and Taylor, 1993; De Boeck et al., 2001). Zinc uptake occurs via the zinc transporter ZIP proteins (ZRT, IRT-like protein) and via the epithelial calcium channel (ECaC), competing in the latter case with Ca^2+^ for uptake (Bury et al., 2003; Zhao et al., 2014). Cadmium can be taken up through the divalent metal transporter (DMT1), and similar to Zn^2+^, it can compete with Ca^2+^ for the uptake via the ECaC, affecting Ca^2+^ homeostasis (Bury et al., 2003; McGeer et al., 2011; Komjarova and Bury, 2014).

One of the earliest findings in ecotoxicology is that temperature can affect tolerance to trace metals in ectotherms (Cairns et al., 1975). Changes in water temperature can occur as a consequence of anthropogenic activities or naturally, e.g., due to the day/night cycle or seasonal changes. The fish’s body temperature is dependent on the ambient temperature, which is one of the most important physical factors for aquatic ectotherms (Brett, 1971). Fish can respond to this alteration physiologically, via thermal adaptation and via behavioral thermoregulation (Ohlberger et al., 2008; Ward et al., 2010). Temperature can influence metabolism, osmoregulation, reproduction, and behavior (Fiess et al., 2007; Crossin et al., 2008; Vergauwen et al., 2010). Generally for metals, elevated temperatures above the optimal temperature range for the organism, tend to enhance toxic effects of metals (Sokolova and Lannig, 2008). Furthermore it has been suggested that an increased metabolic rate at elevated temperatures may contribute to metal accumulation (Sokolova and Lannig, 2008). For instance, in several fish such as Nile tilapia (*Oreochromis niloticus*), zebrafish (*Danio rerio*) and gilthead sea bream (*Sparus aurata*) a higher accumulation of Zn (Karakoc and Dincer, 2003; Guinot et al., 2012), Cd (Vergauwen et al., 2013), and Cu (Guinot et al., 2012) was reported with increasing temperature. Nevertheless, at higher temperatures, also depuration processes might be facilitated (Carvalho et al., 2004; Carvalho and Fernandes, 2006). Several studies in fish, have reported that with increased temperature, also the toxicant accumulation (e.g., mercury, cadmium, and arsenic) and depuration increased (Hodson and Sprague, 1975; McGeachy and Dixon, 1989; Nichols and Playle, 2004). In addition studies on rainbow trout (*Oncorhynchus mykiss*), brown trout (*Salmo trutta*), and stone loach (*Noemacheilus barbatulus*) showed that at higher temperatures the elimination rates of cesium (Cs) and Cd increased (Douben, 1989; Ugedal et al., 1992; Cocchio et al., 1995; Nichols and Playle, 2004).

Fish have defensive mechanisms involved in both essential metal-homeostasis and metal sequestration. Metallothioneins (MT) are low molecular weight metal binding proteins that are involved in the maintenance of Zn and Cu homeostasis (Hamilton and Mehrle, 1986; Coyle et al., 2002; De Boeck et al., 2003). Metallothionein synthesis, although mainly linked with metal exposure, can be affected by other factors such as temperature (Van Cleef-Toedt et al., 2001; Guinot et al., 2012). For instance higher levels of MT proteins were found in sea bream liver when exposed to 30°C in comparison to fish exposed at 22 or 27°C (Guinot et al., 2012). Similarly also in Nile tilapia MT levels increased as water temperature (20, 24, 28, or 32°C) increased (Abdel-Tawwab and Wafeek, 2014).

Although there are several studies describing the effects of single metals and temperature in fish (Abdel-Tawwab and Wafeek, 2017; Braz-Mota et al., 2017; Li et al., 2020), it is difficult to find information on metal mixtures combined with different temperature scenarios. In this study, common carp (*Cyprinus carpio*) were exposed to a sublethal metal mixture of Cu, Zn, and Cd using nominal metal concentrations reflecting the 10% of the 96 h-LC_50_ (the concentration that is lethal to 50% of the population in 96 h) previously determined in our lab by Delahaut et al. (2020) in fish kept at 20°C. The used nominal concentrations were Cu: 0.08 μM; Cd: 0.02 μM and Zn: 3 μM which represents a mixture of metals at similar toxicity levels for each metal. Moreover, in Flanders (the Belgian region where this study was conducted), the limits for these metals are set to 0.11 μM for Cu and 0.30 μM for Zn, whereas for dissolved Cd in rivers and lakes limits range (according to the water hardness) are between 0.004 to 0.013 μM (or 0.45 and 1.5 μg/L) (VLAREM II, 2010). However, these limits can easily be exceeded as shown by Michiels et al. (2017), who reported dissolved metal concentrations up to 0.95 μM, 0.05 μM, 52 μM for Cu, Cd, and Zn, respectively, in two different rivers. In addition a more recent study reported values ranging, from 0.02 to 0.04 μM for Cu, from 0.74 to 52 μM for Zn, and from 0.004 to 0.06 μM for Cd over five different locations in Belgium (Delahaut et al., 2019). Therefore the waterborne metal concentrations used in this study can be considered as environmentally relevant in Flanders.

The main aim of the present study was to understand to which extent different temperatures can affect metal mixture toxicity in common carp. This question will be answered by investigating metal bioaccumulation and MT gene expression as one of the defensive mechanisms in fish exposed to metals. Moreover changes in electrolyte levels, together with changes in the expression of genes involved in metal uptake and ion-homeostasis, that included CTR1, NHEs, Na^+^/K^+^-ATPase, H^+^-ATPase were assessed. Finally, considering their ecological importance, integrative measures such as the hepatosomatic index (HSI) and condition factor (CF) were measured as well.

Based on previous results obtained in our lab (Castaldo et al., 2020b; Delahaut et al., 2020), we hypothesized on the one hand a fast Cu and Cd bioaccumulation, and on the other hand a delayed Zn accumulation occurring only after 1 week of exposure. Moreover we hypothesize that the presence of metals would trigger the response of MTs in order to mitigate possible deleterious effects. Concerning the electrolyte levels, as mentioned above, due to shared uptake routes between metal and electrolyte ions, a fast Na drop is hypothesized, together with a smaller Ca decrease. Furthermore, considering that a lower temperature leads to lower metabolic activity, we hypothesize that more pronounced effects would occur in fish exposed at 20°C than in fish exposed at 10°C. Finally, considering that the slope of the dose-response curves was similar for the metal ions (Delahaut et al., 2020) and that they were so steep that all but the 100% 96 h-LC_50_ could be considered sublethal, we anticipated that this mixture would remain sub-lethal.

## Materials and Methods

### Experimental Model

Juvenile common carp, were obtained from Wageningen University and Research (Netherlands). Fish were kept in 1000 L aquaria, filled with EPA (Environmental Protection Agency) medium hard water (Weber, 1991) at 20°C with a photoperiod of 12 h light and 12 h dark for several months. Prior to the start of the experiment fish were housed in three 200 L polyethylene tanks (PE) filled with EPA medium-hard water. EPA water was reconstituted using four different salts (VWR Chemicals): NaHCO_3_ (1.1427 mM), CaSO_4_.2H_2_O (0.35 mM), MgSO_4_.7H_2_O (0.5 mM), KCl (0.05 mM) using deionized tap water (Aqualab, VWR International, Leuven, Belgium). Aeration and a biofilter were added to maintain water quality. Fish were divided into two series and kept in two different climate chambers (Weiss Technik, Belgium). The first series of fish was kept at 20°C while in the second series the temperature of the climate chamber was reduced by 1°C degree every 3 days until 10°C was reached. The fish were acclimatized for at least 2 weeks at the desired temperatures prior to the start of the experiment. Fish were fed with commercial food (Hikari^®^ Staple^TM^, Klundert, Netherlands) (minimum guaranteed crude protein and fat are 34% and 3%, respectively) *at libitum* once a day for the whole acclimation period. At the beginning of the experiment, in order to give the same amount of feed to the two experimental series, an amount of food representing the 2% of the biomass was given to both the experimental series. This amount was adjusted once during the final weeks as the number of fish per tank decreased. After approximately 5–10 min food leftover was removed from each tank in order to reduce at minimum the metal absorption to the feed and consequent metal ingestion. Experimental methods complied with regulation of the Federation of European Laboratory Animal Science Associations and were approved by the Local Ethics Committee, University of Antwerp (Permit Number: 2015-94 Project 32252).

### Experimental Set Up

The same experimental set up and sampling method was used for both the experimental series, which ran in parallel. Experimental animals (*N* = 100 for each series) were sacrificed in order to collect samples. The fish, 8 months old (length = 55 ± 9 and 57 ± 5 mm; weight = 2.7 ± 1.1 and 2.5 ± 0.6 g, respectively, at 10 and 20°C), were divided between the two thermal exposure series (10°C and 20°C) in control and treatment tanks. Exposure tanks, for each series, consisted of three tanks for control and three PE tanks for the treatment. All the tanks were filled with 150 L of EPA medium-hard water as described above. Water parameters such as pH (8.2 ± 0.2) and conductivity (308 ± 2.5 μS/cm), were measured by the HQ30D Portable Multi-meter (Hach, United States). The dissolved organic carbon (DOC) (0.58 ± 0.23 ppm) was determined following the non-purgeable organic carbon (NPOC) protocol with the high-temperature combustion, using non-dispersive infrared (HTC – NDIR). In each tank, oxygen was provided with an air stone. Water quality was ensured by a biofilter and checked daily. Water was changed when necessary in order to avoid the accumulation of ammonia and other waste products. Water samples were collected before and after water changes to ensure metal concentration stability with the 7700x ICP-MS (Agilent Technologies, Inc., Santa Clara, CA, United States). The measured control total metal concentrations in the 10°C and 20°C series were, respectively, 0.01 ± 0.001 and 0.01 ± 0.002 μM for Cu, whereas Zn and Cd remained below the quantification limit (BMQL). In the treatment during the experiment the total metal levels were for the 10°C series: Cu 0.08 ± 0.02 μM, Zn 2.70 ± 0.42 μM, and Cd 0.02 ± 0.003 μM. For the 20°C series: Cu 0.08 ± 0.02 μM, Zn 2.32 ± 0.35 μM, and Cd 0.02 ± 0.005 μM. Metal speciation for the two experimental series, calculated with the VMinteq software, using measured water parameters is shown in the Supplementary Tables 1, 2. No abnormal fish behavior was observed during the whole experiment.

### Condition Indices

Hepatosomatic Index was calculated according to the formula HSI = LW/BWx100, where BW is the body weight in g and LW is the total liver weight in g. The CF was calculated as CF = BW/L^3^ where L = fish length in cm (Bervoets et al., 2009).

### Metal Accumulation and Electrolyte Levels in the Tissues

At each sampling day (day 1, 7, 14, 21, and 27) ten fish from each treatment and each experimental series, were euthanized with an overdose of MS222 buffered with sodium bicarbonate (pH 7.0, ethyl 3-aminobenzoate methane-sulfonic acid, 300 mg/L, Acros Organics, Geel, Belgium). A muscle sample ≃ 22.4 ± 7.7 mg dry weight (dw) was cut near the caudal fin, the 1st and 4th gill arch of both left and right side were dissected and pooled per two fish to obtain sufficient tissue. The liver and the brain were collected and pooled per two fish. In addition the remaining carcasses per treatment and per sampling day were collected and pooled (two per sample) to have an overview of the whole body accumulation. The wet weight of the sampled tissues was recorded and the samples immediately frozen in liquid nitrogen and stored at −80°C. Metal and electrolyte levels in gills, liver, muscle, and brain were determined in five samples from each tissue (according to the pooled number of samples) at each sampling time and for each treatment. Samples, reference material (SRM-2976, Mussel tissue, National Institute of Standards and Technology, Gaithersburg, MD, United States) and blanks were collected in pre-weighed Eppendorf bullet tubes, dried for 48 h at 60°C and cooled in a desiccator for 2 h. Subsequently the dry weight of the samples was recorded with a precision scale (Sartorius SE2, ultra microbalance). The digestion process was done as described by Reynders et al. (2006a) and Blust et al. (1988). Briefly, after a sample digestion of 12 h at room temperature using 69% concentrated HNO_3_ and three microwave steps, H_2_O_2_ was added to destroy the fat tissue and a further microwave digestion took place. Carcass samples (*N* = 5), collected in pre-weighed 50 ml Falcon tubes were processed similarly. The samples were digested using a hot block (Environmental Express, Charleston, SC, United States) for 30 min at 100°C. At the end of the digestion process, all the samples were diluted using ultrapure Milli-Q (MQ), to reach a final acid volume concentration between 1 and 3%.

Metal and electrolyte levels for the liver and carcasses were expressed not only as nmol/g dw or μmol/g dw, but also as nmol/tissue dw or μmol/tissue dw (calculated by multiplying the reported concentrations by the dw in g of the pooled tissue of interest) in order to take into account the effect of the temperature on liver size and the natural growth of the organism.

Metal and electrolyte levels were determined, respectively, using a 7700x ICP-MS (Agilent Technologies, Santa Clara, CA, United States) and an iCAP 6300 Duo (Thermo Fisher Scientific, Waltham, MA, United States). Results obtained with the above instruments, refer to the total element, without considering the charges. Therefore, charges in the manuscript were added only when relevant for the discussion.

### RNA Extraction and Real Time PCR

The second and the third gill arch of individual fish and an aliquot of the pooled liver samples of two fish were used for total RNA extraction and gene expression. The RNA was extracted according to the manufacturer protocol from ∼ 50 mg of tissue using Trizol (Invitrogen, Merelbeke, Belgium). RNA quantity and purity was determined with nano-Drop spectrophotometry (NanoDrop Technologies, Wilmington, DE, United States), whereas the integrity was assessed with a 1% agarose gel. The cDNA was synthesized according to RevertAid H minus First strand cDNA synthesis kit protocol (Thermo Fisher Scientific, Fermentas, Cambridgeshire, United Kingdom). According to the OD260/OD280 nm absorption ratio (higher than 1.8), four samples were selected and used for qPCR. The Real-time PCR was performed using the Brilliant III Ultra-Fast QPCR Master Mix (Agilent) for the Mx3000P QPCR System. The assay was performed following the Brilliant III Ultra-Fast QPCR Master Mix (Agilent) protocol for Agilent Mx3005P QPCR system in a final reaction volume of 20 μl. The reaction mixture contained 10 μl of Brilliant III Ultra-Fast QPCR Master Mix, 5.7 μl of sterile water, 500 nM of each primer, 0.3 μl of reference dye and 5 ng of cDNA. The contamination of reagent was assessed including the “no template” control (e.g., sterile water) in the analysis. The general experimental run protocol as described by Shrivastava et al. (2017), consisted of a denaturation program (3 min at 95°C), an amplification and quantification program repeated 40 times (15 s at 95°C, 20 s at 60°C) and a melting curve program (60–95°C). Oligonucleotide primers were, β-actin (Wu et al., 2014), eEF (Sinha et al., 2012), H^+^-ATPase (Sinha et al., 2016), Na^+^/K^+^-ATPase (Castaldo et al., 2020b), Na^+^/H^+^ exchanger (Castaldo et al., 2020b), CTR1 (Castaldo et al., 2020a), and metallothionein (Reynders et al., 2006b). Quantification cycles (Cq) values were automatically calculated on the log curve for each gene with MxPro QPCR software (Agilent Technologies, Waldbronn, Germany). The stability of the reference genes was tested by two-ways ANOVA both in liver and in the gills. The presence of unique PCR product was assessed by means of the melting curve and the PCR product was verified on agarose gel. The primer efficiency was determined based on the slope of the standard curve. And the relative gene expression determined by means of the 2^–ΔΔCt^ method (Livak and Schmittgen, 2001). More primers information (e.g., sequence and efficiency) are given in Supplementary Table 3.

### Statistical Analysis

All data are presented as mean values ± S.D. For the statistical analyses, normality of the data was tested with the Shapiro–Wilk test and the homogeneity of the variance with the Levene test. If normality assumption was not met, data were log(y) or sqrt(y) transformed. Three-way analyses of variance (ANOVA), followed by Tukey test, were performed for the obtained data. The independent variables included in the analysis were temperature, dose and time. Data were considered statistically significant when *p*-value < 0.05. All statistical tests, with the exception of the Levene test, which was performed with R 3.6.0 software, were carried out with GraphPad Prism version 8.02 for Windows (GraphPad Software, La Jolla, CA, United States). According to Custer et al. (2000), for metal concentrations below the minimum quantification limit, a value of MQL/2 was assigned. If >50% of the observations were BMQL, no statistical tests were conducted. Data presented in the Supplementary Information, such as curve fitting the metal bioaccumulation as function of time, were analyzed using the same software.

## Results

### Hepatosomatic Index and Condition Factor

**FIGURE 1:**
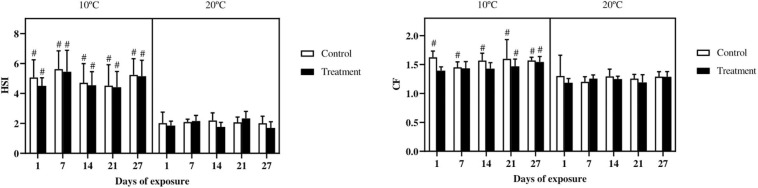
Hepatosomatic index (HSI) and condition factor (CF), in common carp (*Cyprinus carpio*) exposed to a mixture of Cu, Zn, and Cd for 27 days both at 10°C and 20°C. Mean ± SD, *N* = 10. The hash (#) indicates differences between the same groups (both control and metal treated) at different temperatures (*p* < 0.05).

The HSI showed a marked difference, with higher values in all the groups of fish exposed at 10°C as compared with fish exposed at 20°C; no differences were observed between controls and treatments (Figure 1). The CF in fish exposed at 10°C was higher in all the control groups as compared with the same groups at 20°C. From day 21 onward, also the treatment groups at 10°C showed higher values as compared to fish exposed at 20°C. However, no differences were observed between control and metal treated groups (Figure 1).

### Metal Bioaccumulation

#### Metal Accumulation in the Gills

**FIGURE 2:**
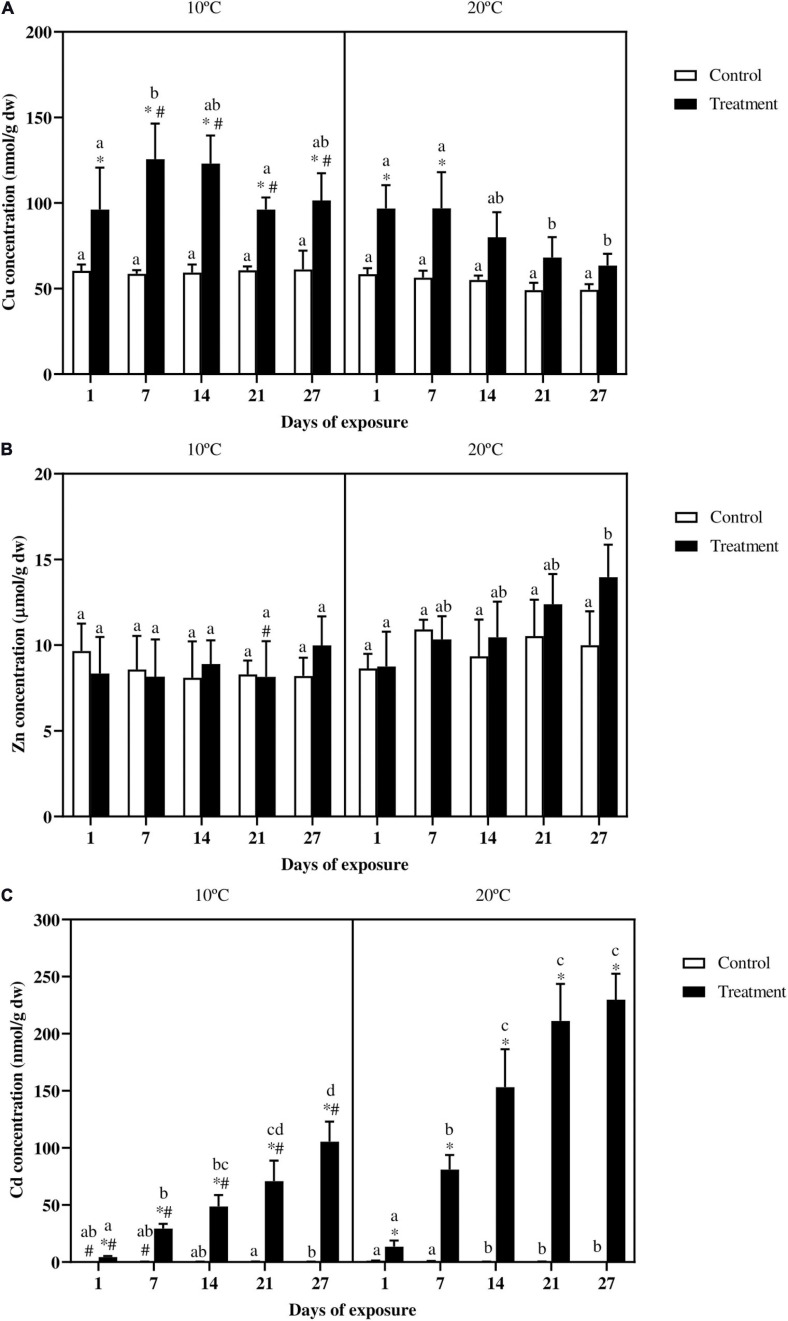
Gills **(A)** Cu (nmol/g dw), **(B)** Zn (μmol/g dw), and **(C)** Cd (nmol/g dw) accumulation in common carp exposed to a mixture of Cu, Zn, and Cd for 27 days both at 10°C and 20°C. Mean ± SD, *N* = 5. Mean + SD. Asterisks (^∗^) indicate differences between control and metal treatment at each sampling day, the hash (#) indicates differences between the same groups (both control and metal treated) at different temperatures, and lowercase letters indicate significant differences of the same group among sampling days at each temperature (*p* < 0.05).

For fish exposed at 10°C Cu content in fish gills (Figure 2A) was higher in the treatment compared to the control for the whole duration of the experiment. In fish exposed at 20°C Cu content increased significantly in the treatment at day 1, and peaked at day 7. After that, from day 14 onward, a clear decreasing trend can be observed with values in metal exposed fish reaching control levels by the end of the experiment. Regarding the effect of the temperature, Cu levels in the control groups were stable within and between temperatures, whereas in the metal exposed groups from day 7 onward, Cu content was always higher at 10°C. Accumulation curves for Cu (Supplementary Figure 1) showed a steady-state pattern for fish exposed at 10°C, whereas for fish exposed at 20°C a clear linear decreasing trend can be observed.

Concerning Zn accumulation, no statistical differences were observed between control and metal exposed groups in the analyzed tissues (Figure 2B). Zinc levels, both in the control and treatment groups remained constant in fish exposed at 10°C, whereas in fish exposed at 20°C, the treatment group accumulated more Zn at day 27 as compared to the beginning of the experiment. Regarding the effect of the temperature, the only difference occurred in the treatment group at day 21 for fish exposed at the lower temperature, which showed lower metal levels as compared with the same group at 20°C. Even though no differences occurred between controls and metal treatments, Zn accumulation curves showed a linear trend, which was more marked at 20°C (Supplementary Figure 1).

Cadmium accumulation in the gills (Figure 2C) was always higher in the metal exposed fish as compared with the control groups in both the temperature scenarios. Over time, at 10°C the control values showed some variation, with a difference occurring between day 21 and 27, whereas for the treated groups a continuous increase can be observed from day 1 onward reaching the highest Cd content at day 27. In fish exposed at 20°C the control groups at day 1 and 7 slightly varied compared to the same groups at different sampling days. In the treatment group, Cd content at day 14 was significantly higher compared with day 7, which was in turn higher compared to day 1. No differences occurred between the treatment groups at day 14, 21, and 27. In addition fish exposed to the metal mixture at 10°C accumulated less Cd compared to fish exposed at 20°C.

Cadmium accumulation showed an almost linear increase in both the exposure scenarios, without reaching a steady-state (Supplementary Figure 1). As suggested also by the accumulation rates this was, in particular, true for fish exposed at 10°C. The accumulation rates for fish exposed at the lower temperature remained constant through the whole experiment, whereas in fish exposed at 20°C, the accumulation rates by the end of the experiment were around the 30% lower compared to the large increase at day 1 (Supplementary Table 6).

#### Metal Bioaccumulation in the Liver

Copper concentration in fish liver (Figure 3.1-A) did not show differences between control and metal exposed fish in either of the temperature scenarios, with the exception of the treatment group at day 14 at 20°C. In fish exposed at 10°C, the Cu concentration for the control groups at day 1 and 7, and for the treatment groups at day 1, 7, 14, and 21 is lower as compared with the same groups of fish exposed at 20°C. Regarding the differences over time, even though a slight increasing Cu trend can be observed at 10°C, no differences occurred between the same treatments at different sampling points. In fish exposed at 20°C the only difference can be observed between the control groups at day 1 and 14. Because liver sizes were considerably larger at 10°C compared to 20°C (see previous paragraph on HSI), we also reported absolute metal levels as metal content in the total liver (Figure 3.2). For Cu content in the total liver (Figure 3.2-A), no differences occurred between control and treatment in both the thermal profiles. However, in fish exposed at the lower temperature a more marked increasing trend can be observed with Cu levels becoming significantly higher in the final days of the experiment as compared to day 1. Moreover both the control and metal exposed groups at day 27 showed higher metal levels as compared with the same groups at 20°C.

**FIGURE 3:**
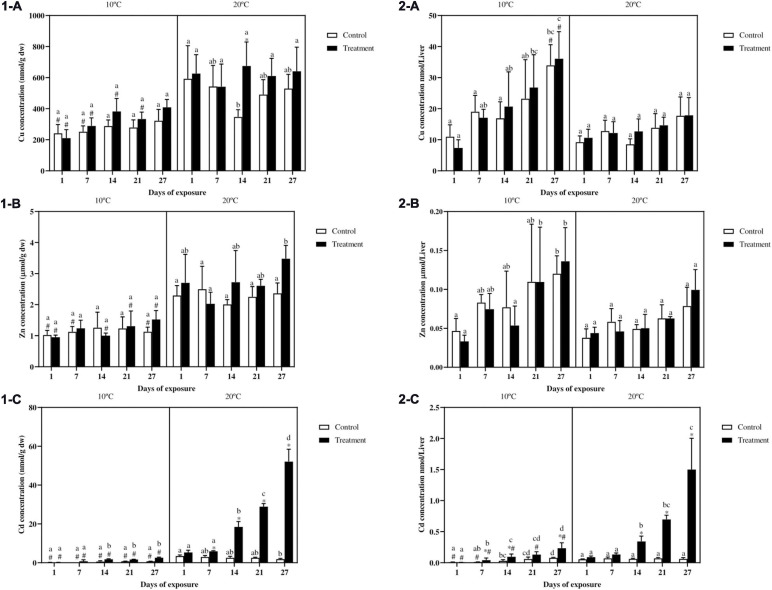
Cu **(1-A)**, Zn **(1-B)**, Cd **(1-C)** expressed as nmol/g dw or as μmol/g dw in the liver and Cu **(2-A)**, Zn **(2-B)**, Cd **(2-C)** expressed as total amount in nmol or μmol per liver in common carp exposed to a mixture of Cu, Zn, and Cd for 27 days both at 10°C and 20°C. Mean + SD, *N* = 5. Mean ± SD. Asterisks (^∗^) indicate differences between control and metal treatment at each sampling day, the hash (#) indicates differences between the same groups (both control and metal treated) at different temperatures, and lowercase letters indicate significant differences of the same groups among sampling days at each temperature (*p* < 0.05).

Zinc concentration in fish liver (Figure 3.1-B) showed no differences between control and metal treated fish in both the temperatures. For fish at 10°C a constant Zn concentration can be observed, showing lower values for nearly all the groups compared to fish exposed at 20°C. In fish exposed at 20°C the only difference over time can be observed between the treatment groups at day 7 and 27. When considering the total Zn content in the whole liver (Figure 3.2-B) similar to what observed for Cu, an increasing trend over time can be noticed in fish exposed at 10°C, with both the control and treatment groups at the end of the experiment showing higher Zn values as compared with the same groups at day 1. No differences were observed between control and metal treated fish as well as for the differences between the two temperature scenarios.

Liver Cd concentration (Figure 3.1-C) showed no differences between control and treatment for fish exposed at 10°C, whereas in fish exposed at 20°C, higher Cd values in the treatment can be observed from day 7 onward. In addition the cadmium concentration in fish exposed to the low temperature was always lower as compared with fish exposed at 20°C. Regarding differences in time, for both the exposure scenarios, higher values were noticed in the treatment groups from day 14 onward. Cadmium content in the whole liver (Figure 3.2-C) showed a similar trend to Cd expressed as nmol/g dw (Figure 3.1-C). However, in fish exposed at 10°C, the content in the treatment groups at day 7, 14, and 27 were significantly higher compared to the controls. Furthermore in fish exposed at 20°C, differences between control and treatment became significant from day 14 onward. Differences according to the temperature can be observed in almost all the groups with higher values in fish exposed at 20°C. At both temperatures, differences between the metal treated groups became more evident from day 7 and 14 onward, respectively, reaching the highest values by the end of the experiment.

#### Metal Bioaccumulation in the Other Tissues

Metal levels in the brain (Supplementary Table 10), muscle (Supplementary Table 11), and in the remaining carcasses (Supplementary Tables 12, 13) are shown in the Supplementary Information. In the brain (Supplementary Table 10) no changes in Cu and Zn concentration were observed for either of the two exposure scenarios. Cadmium concentration in fish expose to 10°C stayed below the detection limit in most of the analyzed samples, whereas in the 20°C scenario, was possible to detect the metal in all the treatment samples from day 14 onward (Supplementary Table 10).

In the muscle (Supplementary Table 11) Cu content showed, in general, no differences between control and treatment groups. Nevertheless an increasing trend can be observed for fish exposed at 10°C, with the highest values reached by the end of the experiment. Differences according to temperature can be observed in both the control and the treatment at day 27, with higher values at 10°C. Regarding Zn, the only difference can be observed between the control group at day 1 in fish exposed to the low temperature, which had higher Zn concentration, compared with the same group in fish exposed at 20°C. Cadmium concentration for fish exposed at 10°C remained below the detection limit for the whole experiment, whereas in fish exposed at 20°C it was possible to detect Cd in a few samples at day 14 and 21, and in all the samples at day 27. For more information see SI-material (Supplementary Table 11).

In the remaining carcasses (Supplementary Table 12), no differences were observed in Cu concentrations (nmol/g dw) between control and treatment groups either at 10°C and 20°C. Moreover the only difference occurring between the two temperatures can be noticed between the metal-exposed groups at day 1. In both the temperature scenarios a decreasing trend, with samples at day 27 showing the lowest Cu levels, can be observed in both the control and the metal treatment. Cadmium concentrations in the carcasses showed a rising trend both at 10°C and 20°C. However, in fish exposed to the low temperature, differences between control and treatment can only be observed by the end of the experiment, whereas in fish exposed to 20°C, they can be observed from day 1 onward. Moreover in fish exposed at 10°C, the treatment group at day 27 accumulated more Cd compared to the same group at day 1. In fish exposed at 20°C the treatment groups at day 14 and 21 accumulated more Cd as compared to the same group at the beginning of the experiment. The only difference between the two exposure scenarios can be observed between the treatment groups at day 21, with fish exposed at the low temperature accumulating less metal.

### Metallothionein Gene Expression

**FIGURE 4:**
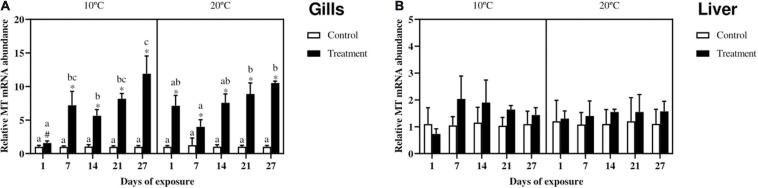
Relative metallothionein (MT) mRNA abundance in gills **(A)** and liver **(B)** of *Cyprinus carpio* exposed to Cu, Zn, and Cd mixtures for 27 days. Mean ± SD, *N* = 4. Asterisks (^∗^) indicate differences between control and metal treatment at each sampling day, the hash (#) indicate differences between the same groups (both control and metal treated) at different temperatures, and lowercase letters indicate significant differences of the same group among sampling days at each temperature (*p* < 0.05).

Gills MT gene expression (Figure 4A) in fish exposed at 10°C increased in the treatment as compared to the control from day 7 onward, whereas in fish exposed at 20°C this increase already started at day one and lasted until the end of the experiment. In both the exposure scenarios almost no differences according to time were noticed, with the exception of the treatment groups at day 27 as compared with the same groups at day 1 and 14 for fish exposed at the low temperature and day 7 for fish exposed at 20°C. Regarding differences between the two temperature scenarios, the only noticeable dissimilarity can be observed between the treatment groups at day 1, with a slower start at 10°C.

In the liver (Figure 4B) no differences can be observed between control and metal-treatment or between the two temperature scenarios.

### Electrolyte Levels

In the gills (Supplementary Table 7), K concentrations of fish exposed both at 10°C and 20°C did not show differences, except for the treatment group at day 21, which showed lower concentrations compared to the same group at day 7 in the 10°C exposure scenario. Regarding Na content the only difference occurred between the control groups at day 1 and 7 and the metal exposed group at day 1, which showed lower values as compared with the same groups exposed at 20°C. No differences were observed for Ca and Mg in either the temperature exposure scenarios.

In the liver, electrolyte concentrations expressed as μmol/g dw are shown in Supplementary Table 8. For K the only differences were observed between the two temperatures, with the control groups at day 1 and 7 and the metal exposed group at day 14 showing lower values in the 10°C scenario. Similarly for Mg and Na all the groups at 10°C showed lower values as compared with fish exposed at 20°C. No differences were noticed for Ca.

In the brain (Supplementary Table 10), the only differences observed in the electrolytes concentrations occurred for K and Na. Regarding K, the day 21 control group of fish exposed at 10°C showed a lower K concentration as compared with the same group exposed at 20°C. Concerning Na, both the control and treatment from day 1 till day 21, showed lower electrolyte concentrations in fish exposed at 10°C, compared with the 20°C scenario.

In the muscle (Supplementary Table 11) no differences were noticed, except for Ca, which showed some variation at the low temperature.

In the remaining carcasses, electrolyte concentrations expressed as μmol/g dw are shown in Supplementary Table 12. For Ca no differences between control and treatment were observed. Furthermore at 10°C, some variation occurred, leading to lower values compared to the start of the experiment, for the treatment at day 21. Similarly also in the 20°C scenario, the control and the treatment groups at day 27 showed lower values as compared with the previous sampling days. Furthermore, in the low temperature scenario, the treatment group at day 21, showed lower values compared to the group at 20°C. Regarding K, Mg, and Na, all three electrolyte showed lower concentrations at 10°C as compared with the 20°C. Moreover a slight decreasing trend, not always significant, could be observed over time both in fish exposed at 10°C and 20°C.

### Expression of Ion Channels

**FIGURE 5:**
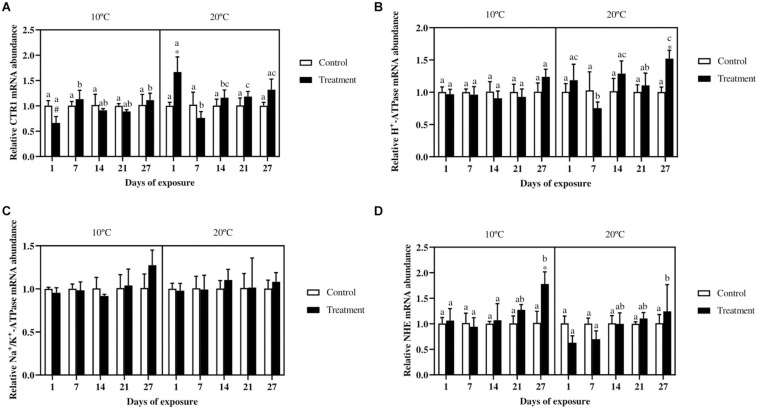
Relative copper transporter 1 **(A)**, H^+^-ATPase **(B)**, Na^+^/K^+^-ATPase **(C)**, and Na^+^/H^+^-exchanger **(D)** mRNA abundance in gills of *Cyprinus carpio* exposed to Cu, Zn, and Cd mixtures for 27 days. Mean ± SD, *N* = 4. Asterisks (*) indicate differences between control and metal treatment at each sampling day, the hash (#) indicate differences between the same groups (both control and metal treated) at different temperatures, and lowercase letters indicate significant differences of the same group among sampling days at each temperature (*p* < 0.05).

The gene expression of the CTR1 in the gills (Figure 5A) did not show differences between the control and metal treatment in fish exposed at 10°C, whereas in fish exposed at 20°C a significant increase can be observed in the treatment group at day 1. In fish exposed to the lowest temperature, the metal exposed groups at day 7 and 27 differ with the same groups at day 1. In the 20°C exposure, the treatment at day 1 showed higher mRNA abundance as compared with the same groups at day 7, 14 and 21. The only differences between the two temperature scenarios occurred for the metal exposed group at day 1, with lower values in fish exposed at 10°C.

For the expression of the H^+^-ATPase (Figure 5B) no differences were observed in fish exposed at 10°C, whereas for fish exposed at 20°C, the treatment group at day 27 showed a higher expression compared with the control group. Regarding the NHE (Figure 5D), the only significant difference between control and treatment can be observed by the end of the experiment in fish exposed at the low temperature. Moreover, the metal treated group at day 27 showed a higher expression compared with the same groups at the previous sampling days. In fish exposed at 20°C no differences occurred between control and treatment, even though an increasing trend for the treatment group over time can be seen. The Na^+^/K^+^-ATPase (Figure 5C) did not show significant differences either for fish exposed at 10°C or at 20°C.

## Discussion

### Condition Indices

The condition factor has frequently been used to express the overall health and wellbeing of fish (Bagenal, 1978; Bolger and Connolly, 1989; Bervoets and Blust, 2003). In the present study, no differences in CF were reported between control and metal exposed fish either at 10°C and 20°C. Similarly, in previous studies using the gudgeon (*Gobio gobio*) or the rainbow trout, no relationship was found between environmental metal levels and CF (Dethloff et al., 2001; Bervoets and Blust, 2003). Nevertheless, a marked difference can be observed between the two temperatures, with the CF being higher in fish exposed at 10°C compared with fish exposed at 20°C. Similar to the present finding, in zebrafish exposed to a chronic thermal scenario, the relative condition factor was lower in fish exposed to higher temperatures (32/34°C) compared with fish kept in cold water (17/22°C) (Pilehvar et al., 2019). Vergauwen et al. (2010) observed a decreased condition factor, which was in line with the energy stores, in zebrafish due to warm temperature (34°C). At lower temperature, the reduced metabolic rate could have resulted in an increased energy availability for growth and thus an increased condition factor (Vergauwen et al., 2010).

The HSI, similar to what observed for the CF, was also affected by the temperature, with higher values in fish at 10°C. This is in agreement with results obtained in rainbow trout, which showed a lower HSI in fish kept at 20°C compared with fish kept at 11°C (Sappal et al., 2015). Furthermore in the common carp, Fine et al. (1996) noticed and increased glycogen content in the liver and an increased HSI in fish kept at low temperature (17°C) as compared to high temperatures (26°C). Moreover, also in cold-acclimatized trout a higher HSI was reported (Bouchard and Guderley, 2003) indicating that energy reserves were stored in the liver (Voss, 1985; Bouchard and Guderley, 2003). On the other hand, this temperature effect was similar in control and metal exposed fish, which suggests that the metal load was not sufficiently high to increase stress and energy demand to an extent that would significantly deplete energy reserves.

### Metal Accumulation Dynamics

In a simplistic view, which ignores the effects of temperature on repair mechanisms, it can be proposed that metal toxicity increase with the increase of temperature. For example, elevated metal toxicity at higher temperatures can be related to an increased uptake rate and thus higher metal levels in the organism. Additionally, elevated metabolic rates result in higher energy demands, and as consequence in elevated ventilation and feeding rates, which can lead to higher exposure to water and food contaminated by metals (Sokolova and Lannig, 2008). However, this is not always the case, as shown by some earlier studies, that reported no or reduced effects of increasing temperature on metal bioaccumulation and toxicity (Lannig et al., 2006; Pereira et al., 2017). Five general toxicity patterns for pollutants have been proposed in a review by Sokolova and Lannig (2008), in which the type I and II are the most common patterns for trace metals. Both in type I and II the toxicity increases with an increasing temperature, however, in the type I the toxicity increases only after a certain temperature threshold is reached.

#### Metal Accumulation in the Gills

One of the initial hypotheses was that metal accumulation, especially for Cu and Cd would occur fast in the gills at both the temperatures, but would be more pronounced in fish exposed at 20°C. Both these assumptions were partially based on previous data obtained in our lab (Delahaut et al., 2020), as well as on the high conditional equilibrium constant, log *K*, for metal-gill binding sites. As reported by Playle (2004), the conditional log *K*, values are 8.6, 7.4, and 5.6 (dm^3^ mol^–1^) for Cd, Cu, and Zn, respectively. Therefore Cd binds about 16 times stronger to gills than Cu and 1000 times stronger than Zn.

Zinc, Cu, and Cd accumulation in the gills, showed different accumulation trends. Zinc did not accumulate at all during the whole exposure period, whereas for Cu and Cd the accumulation started fast but to a different extent in the two thermal scenario. The lack of Zn accumulation in this experiment can be related to the fact that Zn, being one of the most abundant ions in the body, is strictly regulated (Zhao et al., 2014). For example in rainbow trout exposed to 234 μg/l (≃ 3.57 μM) of Zn, the accumulation of Zn started only after 10 days (McGeer et al., 2000). Moreover Lange et al. (2002) found a Zn elevation of 1.4-fold in the treated group with 1000 μg/l (≃ 15 μM) of Zn, relative to the control only after 28 days of exposure. Therefore one can assume that the lack of Zn accumulation is more due to the regulation processes rather than to the fact that it was not taken up. Using data from Van Ginneken et al. (1999), an estimated net Zn accumulation at 3 h of 0.61 μmol/g dw and 0.56 μmol/g dw, respectively, for fish at 10°C and 20°C can be obtained, which falls in the error range of the measurements. This let us assume that the Zn uptake starts quite rapidly and just as rapidly is transferred or eliminated. Nevertheless an inhibitory effect of the metal mixture on Zn uptake can not be excluded. It is known that metals share uptake routes. For instance Cd^2+^ and Zn^2+^, due to their chemical characteristic similarity can compete each other for their uptake (Brzóska and Moniuszko-Jakoniuk, 2001) and for instance a Zn uptake inhibition by Cd was reported by Saibu et al. (2018). Nevertheless it is worth to mention that in this experiment the waterborne Cd and Cu levels were, respectively, at least ≃ 116 and 29 times lower compared to Zn. Therefore the lack of Zn accumulation can not be related solely to inhibited uptake. Regarding Cu, it is possible to observe a counterintuitive trend, with accumulated values at 10°C mostly higher than at 20°C and consistently above control levels. Several studies in different species showed an increased metal content with increased temperature. For instance in zebrafish exposed to Cu and/or Cd, there was higher body metal load when the fish were exposed to warmer temperatures (Pilehvar et al., 2019). The killifish (*Poecilia vivipara*) kept at 28°C accumulated more Cu compared to fish kept at 22°C when exposed to waterborne metal concentrations of 20 μg/l (≃ 0.31 μM). However, at metal concentrations of 9 μg/l (≃ 0.14 μM), no differences occurred between the two thermal exposures (Zebral et al., 2019). Therefore, inherent temperature tolerance, time and metal ion exposure levels also seem to play an important role. The observation of a peak in Cu levels followed by a steady decrease thereafter was rather unexpected considering that previous studies showed a continuous Cu increase or at least a steady state in the gills. For instance, in Nile tilapia, gills exposed to 40 μg/l Cu (≃ 0.63 μM), showed a constant increase in metal load up to day 7, after which the levels stabilized only to increase again from day 14 till day 21 (Monteiro et al., 2005). In European eel, exposed to 12 μg/l Cu (≃ 0.19 μM), the total branchial Cu content was near a steady-state at day 3–6 (Grosell et al., 1998). Furthermore, common carp exposed to 1 μM of Cu for 28 days showed a peak Cu content in the gills, during the first 24 h, followed by slightly lower levels thereafter (Hashemi et al., 2008). The clear decreasing trend in the experiment indicates that the gills are only a temporary target organ for some trace metals (e.g., Cu and Zn), after which they are transferred through the blood stream to other tissues for storage and excretion (Grosell, 2011; Kondera et al., 2014). Interestingly, this seems to be much less the case for the non-essential metal Cd which steadily accumulated at 20°C. Therefore, these transport and depuration processes seem to be more efficient for the essential metals, with Zn levels not increasing at all. Findings in the literature showed that these depuration processes are facilitated at higher temperatures (Carvalho et al., 2004; Carvalho and Fernandes, 2006). So it seems that the depuration processes at 20°C could counteract and even exceed the Cu uptake rates at 20°C, whereas in fish kept at 10°C, the lower metabolic activity slowed down the transfer of the metal into other tissues, resulting in elevated metal loads in the gills. Finally, similar to our observation, such a fast accumulation for Cd was already reported in common carp exposed to a combined metal solution, representing 1/10th of LC_50_/48, of chromium (Cr), nickel (Ni), Cd, and lead (Pb) (≃ 96, 85, 45, and 24 μM of Cr, Ni, Cd, and Pb, respectively, or 5 ppm) (*T* not specified) (Vinodhini and Narayanan, 2008). Similarly also in the juvenile olive flounder (*Paralichthys olivaceus*), exposed to several Cd concentrations, an increasing metal trend was observed in the gills during the whole 30 days Cd-exposure period at *T* = 23°C (Kim et al., 2010). In the present study, temperature showed an effect with lower Cd values in fish exposed at 10°C. Similar findings were already reported in the Japanese eel (*Anguilla japonica*), in which lower Cd levels were reported in the eel exposed at 15°C than at *T* = 25–30°C (Yang and Chen, 1996). In principle the bioavailability of metals might be affected by the temperature, thus at higher temperatures, higher levels of free metal ions might be expected. Nonetheless as shown by empirical data and models (e.g., the biotic ligand model and the free ion activity model), the temperature in environmentally relevant ranges has a minor effect on metal speciation (Luoma, 1983; Bervoets and Blust, 1999; Hassler et al., 2004; Sokolova and Lannig, 2008). Thus as suggested by Sokolova and Lannig (2008), in order to explain the role of the temperature on metal uptake and accumulation, it is important to consider the biological effect of the temperature. Therefore the different trends observed for the different metal accumulations are more likely to be related to the effects of the temperature on the metabolic processes.

In vertebrates several mechanisms are known to be involved in Cu homeostasis, including the CTR1 (Anni et al., 2019). The regulation of the CTR1 is often contradictory and unclear (Boyle et al., 2011) and can be influenced by several metal ions. For instance, in zebrafish gills exposed to 0.016 μM Cu an upregulation of this transporter occurred (Leung et al., 2014). Whereas in sea bream a downregulation of the CTR1 in the intestine was observed in response to a high Cu diet, but not during waterborne metal exposures (Minghetti et al., 2008). In zebrafish liver exposed to 2.5 and 5 mg/l (≃ 20, 40 mM) of Cd, an inhibitory effect on the CTR1 gene was observed (Zhu et al., 2018). Similarly a decreased expression was observed in the liver of zebrafish exposed to 200 μg/l (≃ 1.77 μM) of Cd, with a further decrease observed when fish were exposed at 34°C (Guo et al., 2018). In the present experiment the increase observed at day 1 for fish exposed at 20°C, is counterintuitive from a primary defense mechanism point of view, i.e., a downregulation of the transporter would be expected in order to slow down metal accumulation. Moreover, considering the presence of Cd in the mixture, a decreased CTR1 gene expression could be expected, as demonstrated in zebrafish (Guo et al., 2018; Zhu et al., 2018). It is known that cortisol, which plays a role in the up-regulation of CTR1 gene (Tellis et al., 2012), can increase in response to metal exposure (De Boeck et al., 2001). Therefore, the observed trend may be attributed to an unexpected stressful situation for the organism, such as the metal exposure, which increased temporarily cortisol levels, leading to temporary up-regulation of this gene. Nevertheless further studies are needed to validate this thought.

#### Metal Accumulation in the Remaining Tissues

Metal accumulation in the remaining tissues showed a different pattern between essential metals and non-essential metals. Zinc and Cu levels in the metal exposed groups did not show any significant increase as compared to the control in any of the analyzed tissues. For example, similar findings were already observed for Cu in common carp muscle and in rainbow trout brain (De Boeck et al., 1997; Shaw et al., 2012). This gives an indication that the liver storage capacity and excretion processes were able to cope with the metal concentrations. However, for the non-essential metal Cd, the situation is different suggesting that at lower temperatures the lower metabolic activity can result in less efficient metal transfer, as supported by findings in the remaining carcasses. Secondly this gives an indication of the important role played by the liver, together with the kidney, in protecting more vulnerable organs from metal toxicity as previously observed in different fish species (Brown et al., 1986; Hollis et al., 2001; Reynders et al., 2006a). In the brain the general lack of metal accumulation is reasonable considering that this organ is protected by the blood brain barrier (Evans and Hastings, 1992; Szebedinszky et al., 2001). Nevertheless Cd was detected in all the brain samples of fish exposed at 20°C from day 14 onward, suggesting that temperature and time are important factors for brain. From the obtained results, it seems that for a non-essential metal such as Cd, the temperature effect is marked for most tissues, with higher metal content at 20°C due to the higher metabolism. Our study also highlights that differences in temperature and metabolic rate can have profound effects on condition indices and organ sizes. Therefore in studies taking into account multiple stressors, it is important when interpreting the results to consider the effect of ambient parameters (e.g., temperature) on the organismal fitness (e.g., liver size) which consequently might affect the outcome of the chemical stressor (e.g., metal bioaccumulation and depuration). In this way, dilution effects on metal content are not underestimated.

### Metallothionein Gene Expression

Several studies have pointed out the relationship between metal accumulation and MT levels in different tissues, and the protective role played by this protein toward metal ion toxicity (De Smet et al., 2001; Lange et al., 2002; De Boeck et al., 2003, 2010a). However, to the authors’ knowledge only few studies have been conducted in order to understand the effects of both metals and temperature on MT synthesis in fish.

In the present experiment, the MT mRNA expression was nearly always increased in the gills, but not in the liver which accumulated relatively low metal levels and is known to have higher MT basal level (Hashemi et al., 2008). The increased gene expression in the gills in response to metal accumulation is an appropriate response when metals accumulate. However, further studies assessing post transcriptional events are needed to further validate this thought.

In this experiment, fish kept at 10°C showed a slightly delayed response in the gills. A temperature-dependent induction kinetics of MT was also observed in rainbow trout Zn-exposed hepatocytes, showing that even small changes in temperature (from 9 to 6°C) can have serious impacts on the kinetics of MT efficiency and induction (Hyllner et al., 1989). This can be related to a lower plasma membrane fluidity (Krasne et al., 1971), which in turn affects the ion channel permeability to the metal and subsequent MT mRNA induction (Van Cleef-Toedt et al., 2001).

### Ionoregulation and Electrolyte Levels

It is well known that metals, such as Cu, Zn, and Cd can compete with major electrolyte ions for uptake and thereby alter their physiological levels (Bury et al., 2003; De Boeck et al., 2010b; Grosell, 2011; McGeer et al., 2011). In the present experiment no differences between metal-treated and control fish occurred in any of the analyzed tissues, for either thermal exposure profile. The relatively stable Na levels are surprising considering that gills are one of the most affected tissue as reported in several studies (De Boeck et al., 2001; Grosell and Wood, 2002; Grosell et al., 2002; Mackenzie et al., 2004). Moreover this finding is in disagreement with a previous study from our lab on common carp using comparable metal concentrations and showing a Na loss from the first day of exposure (Castaldo et al., 2020b). However, it is worth to mention that fish in the present work were fed. It has been demonstrated that in rainbow trout, the ingested Na^+^ is absorbed within hours (Smith et al., 1995), thus the steady levels of Na and electrolytes in general might be related to the presence of food. This thought is supported by the few significant changes that occurred, in the expression of genes involved with Na^+^-homeostasis. However, further chronic studies, including not only gene expression data, but also protein levels and enzyme activity are needed to underline the mechanisms involved in this process.

Temperature changes are known to impair osmoregulatory ability in teleosts, such as common carp (Metz et al., 2003) and Mozambique tilapia (*Oreochromis mossambicus*) (Fiess et al., 2007). However, in this experiment, the only trend was that electrolyte ion concentrations (mainly for Na, in the gills and in the brain) were lower at 10°C. For example in the gills, reduced Na levels were observed during the first week of exposure. One has to take into account that fish were acclimatized for at least 2 weeks at 10°C before the start of the experiment, and this is the time that was needed for rainbow trout exposed to cold water to restore the Na^+^ influx to earlier warm temperature levels (Gonzalez and McDonald, 2000). Possibly, this adjustment of Na homeostasis took a slightly longer in common carp.

## Conclusion

Metal toxicity studies over the past years have mainly been conducted on single metal exposures, using optimal temperatures. In the present experiment, the effects of a mixture of metals at two different temperatures on several endpoints were investigated in fish. Temperature had a considerable effect on condition indices, with higher values at 10°C, suggesting that a decreased metabolic rate led to an increased energy availability for growth and energy storage. As initially hypothesized, efficient regulation processes for Zn took place. In fact no accumulation was reported for this metal at all. Moreover, the results observed in the gills, suggested that the depuration and regulation processes for essential metals over a long exposure time were more efficient at 20°C than at 10°C, as demonstrated by the excretion of the initially elevated amount of accumulated Cu. As initially hypothesized, the metal accumulation in the gills triggered the induction of the MT gene in order to mitigate possible deleterious effects caused by metal species. A hypothesis which was not confirmed by the present study was a fast loss of Na due to the metal mixture. Even though the aim of this work was not to investigate the role of feeding, in our case it is likely that the presence of the food masked the effects of metals on the electrolyte losses which we observed in earlier studies with unfed fish. Nevertheless, more studies focusing on the role of food in the ion homeostasis during metal exposure are needed. Overall, the data suggest that common carp is able handle this level of contaminants, at least under the exposure conditions of this experiment. Nonetheless, as suggested by Sokolova and Lannig (2008), more studies on the interaction of metal pollution together with natural stressors more representative of environmental conditions, such as fluctuating temperature and interaction with salinity and CO_2_ on different levels of biological organization are needed to construct models to predict the effects of multiple stressors on ectotherm populations and to determine safety margins in ecological risk assessment.

## Data Availability Statement

The original contributions presented in the study are included in the article/Supplementary Material, further inquiries can be directed to the corresponding author.

## Ethics Statement

The animal study was reviewed and approved by the University of Antwerp Permit Number: 2015-94 Project 32252.

## Author Contributions

GC and GDB conceived the study and developed the research idea. GC and MP discussed the experimental design and conducted the experiment. GC, MP, and LA managed the fish facility. GC and LA analyzed the samples. All authors commented, read and approved the previous and final versions of the manuscript.

## Conflict of Interest

The authors declare that the research was conducted in the absence of any commercial or financial relationships that could be construed as a potential conflict of interest.
