# Supplementary material for: Temperature Effects During a Sublethal Chronic Metal Mixture Exposure on Common Carp (Cyprinus carpio)
Source: Front Physiol. 2021 Mar 16;12:651584. doi: 10.3389/fphys.2021.651584 (PMC8009323; doi:10.3389/fphys.2021.651584)
Supplement: Supplementary file 1 [file Data_Sheet_1.docx]

SI-Fig. 1. Gill Cu, Zn and Cd concentration over time. Dotted lines indicate assumed accumulation during the first day, extrapolating starting values from control values at day 1. The Michaelis-Menten (Y = Vmax*X/(Km + X)) curves and lines (Y= YIntercept + X*Slope) were fitted using measured metal concentration in the tissue.

SI-Table 1. Chemical speciation in the exposure media, for fish exposed at 10 ºC calculated using nominal salt concentrations and measured metal levels with the equilibrium speciation code VMinteq.

| Component | Concentration (mol/l) | % of total concentration | Species name |
| --- | --- | --- | --- |
| Cu^2+^ | 2.9318E-09 | 3.665 | Cu^2+^ |
|  |  | 6.988 | CuOH^+^ |
|  |  | 0.027 | Cu(OH)_3_^-^ |
|  |  | 0.561 | Cu(OH)_2_ (aq) |
|  |  | 0.314 | CuSO_4_ (aq) |
|  |  | 86.674 | CuCO_3_ (aq) |
|  |  | 0.134 | CuHCO_3_^+^ |
|  |  | 1.635 | Cu(CO3)_2_^2-^ |
| Zn^2+^ | 1.6675E-06 | 61.760 | Zn^2+^ |
|  |  | 0.035 | Zn(CO_3_)_2_^2-^ |
|  |  | 2.429 | ZnOH^+^ |
|  |  | 14.988 | Zn(OH)_2_ (aq) |
|  |  | 5.326 | ZnSO_4_ (aq) |
|  |  | 0.042 | Zn(SO_4_)_2_^2-^ |
|  |  | 14.274 | ZnCO_3_ (aq) |
|  |  | 1.133 | ZnHCO_3_^+^ |
| Cd^2+^ | 1.6550E-08 | 82.749 | Cd^2+^ |
|  |  | 0.264 | CdOH^+^ |
|  |  | 0.286 | CdCl^+^ |
|  |  | 7.249 | CdSO_4_ (aq) |
|  |  | 0.092 | Cd(SO4)_2_^2-^ |
|  |  | 1.521 | CdHCO_3_^+^ |
|  |  | 7.791 | CdCO_3_ (aq) |
|  |  | 0.040 | Cd(CO_3_)_2_^2-^ |

SI-Table 2*.*  Chemical speciation in the exposure media, for fish exposed at 20 ºC calculated using nominal salt concentrations and measured metal levels with the equilibrium speciation code VMinteq.

| Component | Concentration (mol/l) | % of total concentration | Species name |
| --- | --- | --- | --- |
| Cu^2+^ | 2.2136E-09 | 2.767 | Cu^2+^ |
|  |  | 8.839 | CuOH^+^ |
|  |  | 0.020 | Cu(OH)_3_^-^ |
|  |  | 1.626 | Cu(OH)_2_ (aq) |
|  |  | 0.265 | CuSO_4_ (aq) |
|  |  | 84.284 | CuCO_3_ (aq) |
|  |  | 0.131 | CuHCO_3_^+^ |
|  |  | 2.063 | Cu(CO3)_2_^2-^ |
| Zn^2+^ | 1.3287E-06 | 57.273 | Zn^2+^ |
|  |  | 0.054 | Zn(CO_3_)_2_^2-^ |
|  |  | 5.042 | ZnOH^+^ |
|  |  | 13.846 | Zn(OH)_2_ (aq) |
|  |  | 5.332 | ZnSO_4_ (aq) |
|  |  | 0.038 | Zn(SO_4_)_2_^2-^ |
|  |  | 17.049 | ZnCO_3_ (aq) |
|  |  | 1.354 | ZnHCO_3_^+^ |
| Cd^2+^ | 1.5929E-08 | 79.644 | Cd^2+^ |
|  |  | 0.561 | CdOH^+^ |
|  |  | 0.282 | CdCl^+^ |
|  |  | 7.809 | CdSO_4_ (aq) |
|  |  | 0.087 | Cd(SO4)_2_^2-^ |
|  |  | 1.887 | CdHCO_3_^+^ |
|  |  | 9.658 | CdCO_3_ (aq) |
|  |  | 0.064 | Cd(CO_3_)_2_^2-^ |

SI-Table 3. Set of primers (F=forward; R=reverse) designed for common carp using Primer blast (NCBI) or taken from literature and used for gene expression analysis by quantitative RT-PCR. Reference genes: elongation factor 1α (eEF) and β-actin; target genes: H^+^-ATPase, copper transporter 1 (CTR1), Na^+^/H^+^-exchanger (NHE-2) and Na^+^/K^+^-ATPase and metallothionein (MT).

| Gene | Accession number | Primer 5’🡪 3’ | Annealing temperature (°C) | % GC | % efficiency |
| --- | --- | --- | --- | --- | --- |
| eEF | Sinha et al. (2012)  AF485331.1 | F - TGGAGATGCTGCCATTGT  R - TGCAGACTTCGTGACCTT | 58 | 50  50 | 92 |
| β-actin | Wu et al. (2014)  M24113 | F - CGTGATGGACTCTGGTGATG  R - TCGGCTGTGGTGGTGAAG | 62 | 55  61.1 | 96 |
| H^+^-ATPase | Sinha et al. 2016  JX570880 | F - CTATGGGGGTCAACATGGAG  R - CCAACACGTGCTTCTCACAC | 59 | 55  55 | 103 |
| Na^+^/K^+^- ATPase | Castaldo et al. 2020  JX570881.1 | F - ATGGGTCGTATCGCCACTCT  R - CCAGGAAGACAGCAACACCA | 55 | 55  55 | 104 |
| NHE-2 | XM_019098528 | F - CACACAAGCTTACGACGCAG  R - TCCAGTGTGAACGAGTCTCC | 57 | 55  55 | 107 |
| CTR1 | XM_019104458.1 | F - TCATCAACACACCAGGAGGA  R - AATAGGAACTCACGGGCGAT | 60 | 50  50 | 97 |
| MT | Reynders et al. 2006 | F - CCAAGACTGGAACTTGC  R - ACGTTGACCTCCTCAC | 60 | 52.9  56.3 | 93 |

| **Copper** | | | | | | | | | | | |
| --- | --- | --- | --- | --- | --- | --- | --- | --- | --- | --- | --- |
|  | **Day 1** | | **Day 7** | | **Day 14** | | **Day 21** | | **Day 27** | |  |
| **10 ºC** | Control 10 ºC | Treatment 10 ºC | Control 10 ºC | Treatment 10 ºC | Control 10 ºC | Treatment 10 ºC | Control 10 ºC | Treatment 10 ºC | Control  10 ºC | Treatment 10 ºC |  |
| **Total metal content**  **(nmol/g dw)** | 60.4 ± 3.67 | 96.19 ± 24.45 | 58.62 ± 2.19 | 125.58 ± 20.8 | 59.37 ± 4.68 | 123.04 ± 16.44 | 60.65 ± 2.32 | 96.19 ± 7 | 61.13 ± 10.99 | 101.48 ± 15.91 |  |
| **Net acc**  **(nmol/g dw)** | / | 35.79 ± 24.45 | / | 66.97 ± 20.8 | / | 63.67 ± 16.44 | / | 35.54 ± 7 | / | 40.35 ± 15.91 |  |
| **% increase** | / | 59.24 ± 40.48 | / | 114.25 ± 35.49 | / | 107.24 ± 27.69 | / | 58.6 ± 11.54 | / | 66.01 ± 26.03 |  |
| **Accumulation rate**  **(nmol g^-1^ dw h^-1^)** | / | 1.49 ± 1.02 | / | 0.4 ± 0.12 | / | 0.19 ± 0.05 | / | 0.07 ± 0.01 | / | 0.06 ± 0.02 |  |
|  | **Day 1** | | **Day 7** | | **Day 14** | | **Day 21** | | **Day 27** | |  |
| **20 ºC** | Control 20 ºC | Treatment 20 ºC | Control 20 ºC | Treatment 20 ºC | Control 20 ºC | Treatment 20 ºC | Control 20 ºC | Treatment 20 ºC | Control  20 ºC | Treatment 20 ºC |  |
| **Total metal content**  **(nmol/g dw)** | 58.37 ± 3.57 | 96.81 ± 13.61 | 56.34 ± 4.09 | 96.85 ± 21.13 | 55.05 ± 2.5 | 79.99 ± 14.62 | 49.05 ± 4.39 | 68.19 ± 11.9 | 49.27 ± 3.25 | 63.44 ± 6.82 |  |
| **Net acc**  **(nmol/g dw)** | / | 38.44 ± 13.61 | / | 40.51 ± 21.13 | / | 24.94 ± 14.62 | / | 19.15 ± 11.9 | / | 14.16 ± 6.82 |  |
| **% increase** | / | 65.85 ± 23.32 | / | 71.91 ± 37.51 | / | 45.29 ± 26.56 | / | 39.04 ± 24.26 | / | 28.74 ± 13.85 |  |
| **Accumulation rate**  **(nmol g^-1^ dw h^-1^)** | / | 1.6 ± 0.57 | / | 0.24 ± 0.13 | / | 0.07 ± 0.04 | / | 0.04 ± 0.02 | / | 0.02 ± 0.01 |  |

**SI-Table 4.** Cu content, net accumulation, percentage of increase and accumulation rate, in fish gills exposed to a metal mixture for 27 days either at 10 ºC or 20 ºC.

| **Zinc** | | | | | | | | | | | |
| --- | --- | --- | --- | --- | --- | --- | --- | --- | --- | --- | --- |
|  | **Day 1** | | **Day 7** | | **Day 14** | | **Day 21** | | **Day 27** | |  |
| **10 ºC** | Control 10 ºC | Treatment 10 ºC | Control 10 ºC | Treatment 10 ºC | Control 10 ºC | Treatment 10 ºC | Control 10 ºC | Treatment 10 ºC | Control 10 ºC | Treatment 10 ºC |  |
| **Total metal content**  **(µmol/g dw)** | 9.65 ± 1.6 | 8.34 ± 2.14 | 8.58 ± 1.97 | 8.16 ± 2.18 | 8.1 ± 2.12 | 8.89 ± 1.39 | 8.29 ± 0.81 | 8.15 ± 2.09 | 8.2 ± 1.06 | 9.98 ± 1.7 |  |
| **Net acc**  **(µmol/g dw)** | / | -1.31 ± 2.14 | / | -0.42 ± 2.18 | / | 0.79 ± 1.39 | / | -0.14 ± 2.09 | / | 1.78 ± 1.7 |  |
| **% increase** | / | -13.59 ± 22.16 | / | -4.87 ± 25.36 | / | 9.76 ± 17.1 | / | -1.72 ± 25.17 | / | 21.66 ± 20.72 |  |
| **Accumulation rate**  **(µmol g^-1^ dw h^-1^)** | / | -0.055 ± 0.09 | / | -0.002 ± 0.01 | / | 0.002 ± 0.004 | / | -0.00028 ± 0.004 | / | 0.003 ± 0.003 |  |
|  | **Day 1** | | **Day 7** | | **Day 14** | | **Day 21** | | **Day 27** | |  |
| **20 ºC** | Control 20 ºC | Treatment 20 ºC | Control 20 ºC | Treatment 20 ºC | Control 20 ºC | Treatment 20 ºC | Control 20 ºC | Treatment  20 ºC | Control 20 ºC | Treatment 20 ºC |  |
| **Total metal content**  **(µmol/g dw)** | 8.64 ± 0.85 | 8.75 ± 2.03 | 10.92 ± 0.56 | 10.34 ± 1.35 | 9.35 ± 2.15 | 10.46 ± 2.09 | 10.52 ± 2.13 | 12.39 ± 1.76 | 9.99 ± 1.98 | 13.97 ± 1.89 |  |
| **Net acc**  **(µmol/g dw)** | / | 0.11 ± 2.03 | / | -0.58 ± 1.35 | / | 1.11 ± 2.09 | / | 1.86 ± 1.76 | / | 3.97 ± 1.89 |  |
| **% increase** | / | 1.33 ± 23.5 | / | -5.32 ± 12.34 | / | 11.88 ± 22.31 | / | 17.72 ± 16.7 | / | 39.78 ± 18.93 |  |
| **Accumulation rate**  **(µmol g^-1^ dw h^-1^)** | / | 0.005 ± 0.08 | / | -0.003 ± 0.008 | / | 0.003 ± 0.006 | / | 0.004 ± 0.003 | / | 0.006 ± 0.003 |  |

**SI-Table 5.** Zn content, net accumulation, percentage of increase and accumulation rate, in fish gills exposed to a metal mixture for 27 days either at 10 ºC or 20 ºC.

**SI-Table 6.** Cd content, net accumulation, percentage of increase and accumulation rate, in fish gills exposed to a metal mixture for 27 days either at 10 ºC or 20 ºC.

| **Cadmium** | | | | | | | | | | |
| --- | --- | --- | --- | --- | --- | --- | --- | --- | --- | --- |
|  | **Day 1** | | **Day 7** | | **Day 14** | | **Day 21** | | **Day 27** | |
| **10 ºC** | Control 10 ºC | Treatment  10 ºC | Control 10 ºC | Treatment  10 ºC | Control 10 ºC | Treatment  10 ºC | Control 10 ºC | Treatment  10 ºC | Control 10 ºC | Treatment  10 ºC |
| **Total metal content**  **(nmol/g dw)** | 0.36 ± 0.04 | 4.46 ± 0.85 | 0.29 ± 0.06 | 29.32 ± 4.09 | 0.31 ± 0.08 | 48.76 ± 9.98 | 0.44 ± 0.15 | 70.87 ± 18.02 | 0.24 ± 0.12 | 105.39 ± 17.68 |
| **Net acc**  **(nmol/g dw)** | / | 4.09 ± 0.85 | / | 29.03 ± 4.09 | / | 48.44 ± 9.98 | / | 70.43 ± 18.02 | / | 105.15 ± 17.68 |
| **% increase** | / | 1127.13 ± 232.98 | / | 10132.13 ± 1426.4 | / | 15566.51 ± 3207.69 | / | 16110.82 ± 4121.01 | / | 43702.71 ± 7349.01 |
| **Accumulation rate**  **(nmol g^-1^ dw h^-1^)** | / | 0.17 ± 0.04 | / | 0.17 ± 0.02 | / | 0.14 ± 0.03 | / | 0.14 ± 0.04 | / | 0.16 ± 0.03 |
|  | **Day 1** | | **Day 7** | | **Day 14** | | **Day 21** | | **Day 27** | |
| **20 ºC** | Control 20 ºC | Treatment  20 ºC | Control 20 ºC | Treatment  20 ºC | Control 20 ºC | Treatment  20 ºC | Control 20 ºC | Treatment  20 ºC | Control 20 ºC | Treatment  20 ºC |
| **Total metal content**  **(nmol/g dw)** | 1.23 ± 0.07 | 13.41 ± 5.49 | 0.84 ± 0.23 | 81 ± 12.73 | 0.28 ± 0.04 | 153.1 ± 33.24 | 0.38 ± 0.16 | 211.2 ± 32.43 | 0.22 ± 0.07 | 229.76 ± 22.85 |
| **Net acc**  **(nmol/g dw)** | / | 12.18 ± 5.49 | / | 80.16 ± 12.73 | / | 152.82 ± 33.24 | / | 210.82 ± 32.43 | / | 229.53 ± 22.85 |
| **% increase** | / | 991.11 ± 446.69 | / | 9549.87 ± 1516.87 | / | 53814.62 ± 11706.42 | / | 55300.74 ± 8507.77 | / | 102685.13 ± 10221.85 |
| **Accumulation rate**  **(nmol g^-1^ dw h^-1^)** | / | 0.51 ± 0.23 | / | 0.48 ± 0.08 | / | 0.45 ± 0.09 | / | 0.42 ± 0.06 | / | 0.35 ± 0.04 |

**SI-Table 7.** Copper, Cd (nmol/g dw), Zn and electrolyte levels (µmol/g dw) in *Cyprinus carpio* gills, exposed to a metal mixture for 27 days. Mean ± SD, N=5, letters indicate significant differences (p < 0.05). Asterisks (*) indicate differences between control and metal treatment at each sampling day, the hash (#) indicates differences between the same groups (both control and metal treated) at different temperatures and lowercase letters indicate significant differences of the same group among sampling days at each temperature (p < 0.05).

| **10 ºC** |  | **Day 1** | | **Day 7** | | **Day 14** | | **Day 21** | | **Day 27** | |
| --- | --- | --- | --- | --- | --- | --- | --- | --- | --- | --- | --- |
|  |  | **Control** | **Treatment** | **Control** | **Treatment** | **Control** | **Treatment** | **Control** | **Treatment** | **Control** | **Treatment** |
| **Gills** | **Cu** | 60.4 ±  3.67  a | 96.19 ±  24.45  a* | 58.62 ±  2.19  a | 125.58 ±  20.8  b*# | 59.37 ±  4.68  a | 123.04 ±  16.44  ab*# | 60.65 ± 2.32  a | 96.19 ±  7  a*# | 61.13 ± 10.99  a | 101.48 ±  15.91  ab*# |
|  | **Zn** | 9.65 ±  1.6  A | 8.34 ±  2.14  a | 8.58 ±  1.97  a | 8.16 ±  2.18  a | 8.1 ±  2.12  a | 8.89 ±  1.39  a | 8.29 ±  0.81  a | 8.15 ±  2.09  a# | 8.2 ±  1.06  a | 9.98 ±  1.7  a |
|  | **Cd** | 0.36 ±  0.04  ab# | 4.46 ±  0.85  a*# | 0.29 ±  0.06  ab# | 29.32 ±  4.09  b*# | 0.31 ±  0.08  ab# | 48.76 ±  9.98  bc*# | 0.44 ± 0.15  a | 70.87 ±  18.02  cd*# | 0.24 ±  0.12  b | 105.39 ±  17.68  d*# |
|  | **Ca** | 520.55 ± 112.99 | 435.11 ±  68.94 | 467.99 ±  73.66 | 517.93 ±  78.04 | 502.93 ±  40.44 | 469.28 ±  53.92 | 489.09 ± 72.32 | 445.4 ±  31.1 | 519.06 ± 84.72 | 480.36 ±  46.63 |
|  | **K** | 275.45 ±  15.17  a | 272.78 ±  16.29  ab | 258.76 ±  13.06  a | 286.29 ±  23.27  a | 268.63 ±  15.13  a | 260.31 ±  27.66  ab | 254.54 ± 6.85  a | 228.87 ±  21.66  b | 263.95 ± 38.01  a | 261.97 ±  15.58  ab |
|  | **Mg** | 48.82 ±  3.89 | 43.26 ±  7.64 | 45.66 ±  2.69 | 48.41 ±  3.42 | 47.84 ±  2.53 | 46.85 ±  5.27 | 46.13 ± 2.33 | 43.08 ±  3.75 | 51.4 ±  4.23 | 48.07 ±  5.13 |
|  | **Na** | 273.93 ±  13.5  # | 252.02 ±  51.48  # | 267.48 ± 1  5.02  # | 289.44 ±  3.92 | 272.2 ±  10.35 | 254.8 ±  32.95 | 269.92 ± 15.08 | 235.97 ±  22.78 | 270.7 ±  41.3 | 269.07 ±  17.93 |
| **20 ºC** |  |  |  |  |  |  |  |  |  |  |  |
| **Gills** | **Cu** | 58.37 ±  3.57  a | 96.81 ±  13.61  a* | 56.34 ±  4.09  a | 96.85 ±  21.13  a* | 55.05 ±  2.5  a | 79.99 ±  14.62  ab | 49.05 ± 4.39  a | 68.19 ±  11.9  b | 49.27 ± 3.25  a | 63.44 ±  6.82  b* |
|  | **Zn** | 8.64 ±  0.85  a | 8.75 ± 2  .03  a | 10.92 ±  0.56  a | 10.34 ±  1.35  ab | 9.35 ±  2.15  a | 10.46 ±  2.09  ab | 10.52 ± 2.13  a | 12.39 ±  1.76  ab | 9.99 ±  1.98  a | 13.97 ±  1.89  b |
|  | **Cd** | 1.23 ±  0.07  a | 13.41 ±  5.49  a* | 0.84 ±  0.23  a | 81 ±  12.73  b* | 0.28 ±  0.04  b | 153.1 ±  33.24  c* | 0.38 ±  0.16  b | 211.2 ± 3  2.43  c* | 0.22 ±  0.07  b | 229.76 ±  22.85  c* |
|  | **Ca** | 582.71 ±  75.13 | 594.55 ±  117.5 | 599.22 ±  86.83 | 529.09 ±  131.76 | 562.74 ±  86.25 | 509.19 ±  45.33 | 505.06 ± 54.15 | 465.49 ±  18 | 516.54 ± 70.33 | 482.83 ±  67.65 |
|  | **K** | 271.97 ±  12.25  a | 253.51 ±  24.55  a | 264.69 ±  11.28  a | 256.44 ±  41.06  a | 267.42 ±  8.11  a | 263.13 ±  18.27  a | 254.84 ±  25  a | 258.22 ±  11.81  a | 254.1 ±  9.45  a | 287 ±  8.76  a |
|  | **Mg** | 48.8 ±  2.92 | 48.2 ±  6.7 | 51.65 ±  3.83 | 45.56 ±  8.35 | 49.56 ±  3.89 | 47.75 ±  3.08 | 44.67 ±  3.71 | 43.8 ±  1.74 | 48.51 ±  2.64 | 48.69 ±  3.79 |
|  | **Na** | 352.72 ±  21.54 | 322.28 ±  28.2 | 333.89 ±  28.32 | 319.97 ±  45.84 | 319.04 ±  21.39 | 297.83 ±  15.55 | 298.28 ± 14.28 | 286.74 ±  18.61 | 318.26 ± 12.28 | 320.06 ±  3.37 |

**SI-Table 8.** Copper, Cd (nmol/g dw), Zn and electrolyte levels (µmol/g dw) in *Cyprinus carpio* liver, exposed to a metal mixture for 27 days. Mean ± SD, N=5, letters indicate significant differences (p < 0.05). Asterisks (*) indicate differences between control and metal treatment at each sampling day, the hash (#) indicates differences between the same groups (both control and metal treated) at different temperatures and lowercase letters indicate significant differences of the same group among sampling days at each temperature (p < 0.05).

| **10 ºC** |  | **Day 1** | | **Day 7** | | **Day 14** | | **Day 21** | | **Day 27** | |
| --- | --- | --- | --- | --- | --- | --- | --- | --- | --- | --- | --- |
|  |  | **Control** | **Treatment** | **Control** | **Treatment** | **Control** | **Treatment** | **Control** | **Treatment** | **Control** | **Treatment** |
| **Liver** | **Cu** | 241.52 ±  56.57  a# | 210.2 ±  55.32  a# | 251.48 ±  38.07  a# | 288.84 ±  52.09  a# | 288.19 ±  39.04  a | 382.2 ±  83.42  a# | 278.87 ± 48.94  a | 333.42 ±  44.93  a# | 322.3 ± 73.07  a | 408.84 ±  51.02  a |
|  | **Zn** | 1.02 ±  0.15  a# | 0.95 ±  0.07  a# | 1.12 ±  0.17  a# | 1.24 ±  0.26  a | 1.26 ±  0.5  a | 1 ±  0.09  a# | 1.23 ±  0.37  a | 1.31 ±  0.49  a# | 1.13 ±  0.15  a# | 1.52 ±  0.29  a# |
|  | **Cd** | 0.186 ±  0.041  a# | 0.192 ±  0.035  a# | 0.151 ±  0.004  a# | 0.775 ±  0.724  a# | 0.585 ±  0.402  a# | 1.754 ±  0.263  b# | 0.708 ± 0.15  a# | 1.644 ±  0.122  b# | 0.736 ±  0.06  a# | 2.561 ±  0.33  b# |
|  | **Ca** | 3.81 ±  1.56 | 4.15 ±  2.76 | 2.98 ±  1.57 | 2.64 ±  0.49 | 2.52 ±  0.76 | 3.6 ±  2.87 | 3.1 ±  1.35 | 3.19 ±  0.89 | 3.6 ±  1.29 | 2.39 ±  0.31 |
|  | **K** | 183.72 ±  8.17  # | 182.08 ±  16.54 | 196.92 ±  6.14  # | 184.7 ±  11.32 | 198.52 ±  15.83 | 177.89 ±  22.83  # | 195.76 ± 10.49 | 199.66 ±  9.38 | 195.83 ± 11.29 | 206.33 ±  4.14 |
|  | **Mg** | 21.8 ±  1.51  # | 24.25 ±  1.93  # | 22.95 ±  1.11  # | 22.63 ±  1.68  # | 23.62 ±  1.49  # | 23.19 ±  1.61  # | 22.96 ±  1.55  # | 24.03 ±  1.43  # | 22.77 ±  1.14  # | 24.9 ±  1.34  # |
|  | **Na** | 55.84 ±  4.16  # | 66.18 ±  3.25  # | 61.82 ±  3.33  # | 60.65 ±  5.47  # | 64.09 ±  9.99  # | 58.5 ±  7.44  # | 57.73 ±  7.57  # | 59.62 ±  4.48  # | 56.94 ±  4.41  # | 61.07 ±  2.98  # |
| **20 ºC** |  |  |  |  |  |  |  |  |  |  |  |
| **Liver** | **Cu** | 592.11 ± 213.79a | 625.83 ± 122.15a | 542.81 ± 136.06ab | 541.13 ± 146.52a | 346.61 ± 46.86b | 674.96 ± 154.56a* | 490.6 ± 96.27ab | 610.33 ± 114.11a | 529.27 ± 92.17ab | 640.53 ± 155.59a |
|  | **Zn** | 2.29 ±  0.32  a | 2.7 ±  0.92  ab | 2.5 ±  0.74  a | 2.03 ±  0.37  a | 2.01 ±  0.15  a | 2.72 ±  1.02  ab | 2.25 ±  0.33  a | 2.61 ±  0.21  ab | 2.36 ±  0.34  a | 3.48 ±  0.43  b |
|  | **Cd** | 3.436 ± 0.5588  a | 5.287 ±  1.1671  a | 2.972 ± 0.7958  ab | 5.867 ± 0  .3411  a* | 2.539 ± 0.7559  ab | 18.435 ± 2.7902  b* | 2.514 ± 0.3299  ab | 28.871 ± 1.6023  c* | 1.839 ± 0.4076  b | 52.065 ± 6.4067  d* |
|  | **Ca** | 6.4 ±  3.8 | 5.04 ±  2.72 | 5.31 ±  2.61 | 3.54 ±  0.35 | 3.56 ±  0.64 | 4.23 ±  0.84 | 3.82 ±  0.23 | 3.9 ±  0.2 | 3.37 ±  0.25 | 4.1 ±  0.39 |
|  | **K** | 229.76 ±  14.68 | 200.98 ±  15.95 | 244.03 ±  56.57 | 213.66 ±  7.57 | 203.21 ±  30.41 | 230.23 ±  19.5 | 223.73 ±  18 | 227.84 ±  9.79 | 218.9 ±  7.81 | 237.01 ±  6.67 |
|  | **Mg** | 33.52 ±  2.25 | 30.66 ±  1.73 | 29.37 ±  4.58 | 32.1 ±  2.28 | 29.81 ±  4.51 | 35.14 ±  2.94  * | 32.74 ±  2.9 | 32.93 ±  1.25 | 31.69 ±  0.92 | 35.16 ±  1.66 |
|  | **Na** | 136.97 ±  14.9  a | 120.52 ±  14.67  a | 111.31 ±  24.7  b | 111.3 ±  9.74  a | 92.07 ±  7.43  b | 118.74 ±  14.56  a | 112.88 ± 8.34  b | 101.56 ±  3.37  a | 95.1 ±  8.71  b | 122.56 ±  8.14  a* |

**SI-Table 9.** Metal and electrolyte levels multiplied by liver dw and expressed as nmol (Cu and Cd) or µmol (Zn and electrolytes) per total liver in *Cyprinus carpio*, exposed to a metal mixture for 27 days. Mean ± SD, N=5. Asterisks (*) indicate differences between control and metal treatment at each sampling day, the hash (#) indicates differences between the same groups (both control and metal treated) at different temperatures and lowercase letters indicate significant differences of the same group among sampling days at each temperature (p < 0.05).

| **10 ºC** |  | **Day 1** | | **Day 7** | | **Day 14** | | **Day 21** | | **Day 27** | |
| --- | --- | --- | --- | --- | --- | --- | --- | --- | --- | --- | --- |
|  |  | **Control** | **Treatment** | **Control** | **Treatment** | **Control** | **Treatment** | **Control** | **Treatment** | **Control** | **Treatment** |
| **Tot**  **Liver**  **(dw)** | **Cu** | 10.97 ±  3.85  a | 7.38 ±  2.61  a | 19.02 ±  5.24  a | 17.08 ±  2.73  ab | 16.88 ±  5.33  a | 20.68 ±  11.16  ab | 23.18 ± 12.61  ab | 26.8 ±  10.56  bc | 33.93 ±  6.68  bc# | 36.08 ±  8.72  c# |
|  | **Zn** | 0.05 ±  0.02  a | 0.03 ±  0.01  a | 0.08 ±  0.01  ab | 0.07 ±  0.02  ab | 0.08 ±  0.05  ab | 0.05 ±  0.03  a | 0.11 ±  0.07  ab | 0.11 ±  0.07  b | 0.12 ±  0.02  b | 0.14 ±  0.04  b |
|  | **Cd** | 0.008 ±  0.003  a# | 0.006 ±  0.001  a# | 0.011 ±  0.002  ab# | 0.041 ±  0.035  b*# | 0.031 ±  0.019  bc | 0.095 ±  0.046  c*# | 0.059 ±  0.032  cd | 0.13 ±  0.047  cd# | 0.077 ±  0.009  d | 0.234 ±  0.087  d*# |
|  | **Ca** | 0.18 ±  0.1 | 0.15 ±  0.12 | 0.22 ±  0.1 | 0.16 ±  0.02 | 0.15 ±  0.07 | 0.16 ±  0.11 | 0.28 ±  0.25 | 0.25 ±  0.09 | 0.38 ±  0.14# | 0.21 ±  0.06 |
|  | **K** | 8.2 ±  1.72  a# | 6.39 ±  1.77  a | 14.85 ±  3.26  ab# | 11.29 ±  3.4  abc# | 11.45 ±  2.23  ab# | 9.44 ±  4.1  ab# | 16.08 ±  7.68  ab# | 15.74 ±  5.17  bc# | 20.84 ±  2.83  b# | 18.68 ±  6.19  c# |
|  | **Mg** | 0.97 ±  0.18  a | 0.85 ±  0.22  a | 1.73 ±  0.35  ab# | 1.38 ±  0.41  ab# | 1.36 ±  0.25  ab# | 1.23 ±  0.51  a | 1.9 ±  0.95  ab# | 1.89 ±  0.61  b# | 2.42 ±  0.33  b# | 2.28 ±  0.84  b# |
|  | **Na** | 2.49 ±  0.56  a | 2.29 ±  0.48  a | 4.64 ±  0.88  b | 3.66 ±  0.85  abc | 3.68 ±  0.68  ab | 3.05 ±  1.09  ab | 4.72 ±  2.21  b | 4.7 ±  1.52  bc# | 6.06 ±  0.84  b# | 5.58 ±  2.02  c |
| **20 ºC** |  |  |  |  |  |  |  |  |  |  |  |
| **Tot**  **Liver**  **(dw)** | **Cu** | 9.23 ±  2.02  a | 10.62 ±  2.79  a | 12.78 ±  3.51  a | 12.16 ±  3.66  a | 8.54 ±  1.75  a | 12.69 ±  4.01  a | 13.84 ± 4.55  a | 14.65 ±  2.62  a | 17.7 ±  6.08  a | 17.87 ±  5.7  a |
|  | **Zn** | 0.04 ±  0.01  a | 0.04 ±  0.01  a | 0.06 ±  0.02  a | 0.05 ±  0.01  a | 0.05 ±  0.01  a | 0.05 ±  0.02  a | 0.06 ±  0.02  a | 0.06 ±  0  a | 0.08 ±  0.02  a | 0.1 ±  0.03  a |
|  | **Cd** | 0.05 ±  0.008  a | 0.089 ±  0.02  a | 0.07 ±  0.021  a | 0.132 ±  0.025  a | 0.061 ±  0.011  a | 0.343 ±  0.086  b* | 0.069 ±  0.015  a | 0.696 ±  0.068  bc* | 0.061 ±  0.02  a | 1.5 ±  0.5  c* |
|  | **Ca** | 0.11 ±  0.06 | 0.06 ±  0.01 | 0.13 ±  0.08 | 0.08 ±  0.01 | 0.09 ±  0 | 0.08 ±  0.02 | 0.11 ±  0.02 | 0.09 ±  0.01 | 0.11 ±  0.03 | 0.12 ±  0.03 |
|  | **K** | 3.76 ±  0.96  a | 3.39 ±  0.49  a | 5.21 ±  0.81  ab | 4.85 ±  1.13  ab | 5 ±  0.98  ab | 4.28 ±  1.05  ab | 6.17 ±  1.16  ab | 5.5 ±  0.52  ab | 7.31 ±  2.03  b | 6.81 ±  1.97  b |
|  | **Mg** | 0.55 ±  0.13  a | 0.52 ±  0.08  a | 0.76 ±  0.09  ab | 0.72 ±  0.13  ab | 0.73 ±  0.14  ab | 0.65 ±  0.16  ab | 0.9 ±  0.16  ab | 0.79 ±  0.07  ab | 1.05 ±  0.27  b | 1 ±  0.27  b |
|  | **Na** | 2.21 ±  0.47 a | 2.03 ±  0.31 a | 2.61 ±  0.48 a | 2.5 ±  0.41 a | 2.27 ±  0.35 a | 2.22 ±  0.61 a | 3.11 ±  0.56 a | 2.45 ±  0.2 a | 3.17 ±  0.88 a | 3.49 ±  0.89 a |

**SI-Table 10.** Copper, Cd (nmol/g dw), Zn and electrolyte levels (µmol/g dw) in *Cyprinus carpio* brain, exposed to a metal mixture for 27 days. Mean ± SD, N=5, letters indicate significant differences (p < 0.05). Asterisks (*) indicate differences between control and metal treatment at each sampling day, the hash (#) indicates differences between the same groups (both control and metal treated) at different temperatures and lowercase letters indicate significant differences of the same group among sampling days at each temperature (p < 0.05).

| **10 ºC** |  | **Day 1** | | **Day 7** | | **Day 14** | | **Day 21** | | **Day 27** | |
| --- | --- | --- | --- | --- | --- | --- | --- | --- | --- | --- | --- |
|  |  | **Control** | **Treatment** | **Control** | **Treatment** | **Control** | **Treatment** | **Control** | **Treatment** | **Control** | **Treatment** |
| **Brain** | **Cu** | 104.14 ±  14.14 | 107.6 ±  4.51 | 103.11 ±  7.33 | 107.77 ±  5.55 | 92.66 ±  10.39 | 96.67 ±  4.29 | 95.07 ±  6.59 | 99.13 ±  11.8 | 96.57 ±  8.21 | 98.59 ±  10.48 |
|  | **Zn** | 1.15 ±  0.22 | 1.34 ±  0.41 | 1.16 ±  0.09 | 1.13 ±  0.06 | 1.06 ±  0.14 | 1.2 ±  0.18 | 1.16 ±  0.1 | 1.15 ±  0.09 | 1.12 ±  0.12 | 1.27 ±  0.22 |
|  | **Cd** | BMQL | BMQL | BMQL | 0.44 | BMQL | 1.45 ±  0.39 | BMQL | 1.05 ±  0.65 | BMQL | 0.84 |
|  | **Ca** | 23.12 ±  14.49 | 146.88 ±  127.58 | 15 ±  6.94 | 119.73 ±  119.8 | 18.7 ±  14.23 | 41.47 ±  39.83 | 28.65 ± 23.89 | 43.93 ±  54.17 | 10.45 ±  0.79 | 27.12 ±  29.39 |
|  | **K** | 338.77 ±  42.82 | 362.76 ±  27.66 | 339.57 ±  14.28 | 341.7 ±  6.37 | 336.82 ±  10.7 | 326.52 ±  11.74 | 332.02 ± 14.8  # | 325.89 ±  26.5 | 327.44 ±  9.8 | 330.77 ±  5.55 |
|  | **Mg** | 31.46 ±  3.83 | 34.08 ±  2.84 | 31.76 ±  1.26 | 32.98 ±  0.61 | 31.79 ±  0.88 | 31.99 ±  1.22 | 30.87 ±  1.1 | 30.28 ±  2.47 | 30 ±  0.99 | 30.9 ±  0.71 |
|  | **Na** | 193.16 ±  29.81  # | 194.44 ±  4.88  # | 192.18 ±  11.36  # | 198.04 ±  10.24  # | 191.66 ±  5.82  # | 183.28 ±  8.9  # | 195.42 ± 6.57  # | 186.81 ±  14.6  # | 189.04 ± 8.69 | 190.53 ±  6.16 |
| **20 ºC** |  |  |  |  |  |  |  |  |  |  |  |
| **Brain** | **Cu** | 88.11 ±  17.26 | 105.1 ±  9.94 | 94.33 ±  8.81 | 96.49 ±  12.19 | 96.25 ±  4.98 | 99 ±  11.19 | 99.24 ± 17.49 | 100.2 ±  2.42 | 89.3 ±  6.09 | 94.12 ±  14.91 |
|  | **Zn** | 0.96 ±  0.14 | 1.04 ±  0.1 | 1.03 ±  0.03 | 1.18 ±  0.13 | 1.08 ±  0.06 | 1.11 ±  0.09 | 1 ±  0.03 | 1.23 ±  0.05 | 1.03 ±  0.04 | 1.14 ±  0.23 |
|  | **Cd** | 1.86 | 3.09 | BMQL | 1.16 | BMQL | 0.9 ±  0.32 | BMQL | 2.01 ±  1.64 | BMQL | 1.05 ±  0.19 |
|  | **Ca** | 65.52 ±  74.85 | 117.64 ±  129.1 | 31.42 ±  21.78 | 140.96 ±  177.67 | 17.1 ±  7.5 | 15.83 ±  3.14 | 16.55 ±  6.61 | 37.37 ±  25.32 | 12.1 ±  2.41 | 18.67 ±  9.29 |
|  | **K** | 341.28 ±  30.1 | 350.8 ±  18.19 | 363.77 ± 18.54 | 355.67 ±  15.92 | 346.7 ±  14.46 | 361.24 ±  14.44 | 347.71 ± 17.63 | 376.42 ±  12 | 348.95 ± 8.32 | 363.76 ±  3.46 |
|  | **Mg** | 29.79 ±  3.18 | 30.74 ±  1.18 | 30.6 ±  1 | 30.28 ±  1.81 | 30.03 ±  1.09 | 30.28 ±  1.33 | 30.06 ±  1.73 | 32.82 ±  1.35 | 30.64 ±  0.86 | 30.95 ±  0.63 |
|  | **Na** | 234.2 ±  7.04 | 237.84 ±  29.63 | 243.27 ±  18.42 | 242.1 ±  16.47 | 235.25 ±  18.35 | 248.41 ±  20.03 | 234.1 ± 14.59 | 253.95 ±  21.82 | 221.93 ± 7.68 | 222.69 ±  9.49 |

**SI-Table 11.** Copper, Cd (nmol/g dw), Zn and electrolyte levels (µmol/g dw) in *Cyprinus carpio* muscle, exposed to a metal mixture for 27 days. Mean ± SD, N=5, letters indicate significant differences (p < 0.05). Asterisks (*) indicate differences between control and metal treatment at each sampling day, the hash (#) indicates differences between the same groups (both control and metal treated) at different temperatures and lowercase letters indicate significant differences of the same group among sampling days at each temperature (p < 0.05).

| **10 ºC** |  | **Day 1** | | **Day 7** | | **Day 14** | | **Day 21** | | **Day 27** | |
| --- | --- | --- | --- | --- | --- | --- | --- | --- | --- | --- | --- |
|  |  | **Control** | **Treatment** | **Control** | **Treatment** | **Control** | **Treatment** | **Control** | **Treatment** | **Control** | **Treatment** |
| **Muscle** | **Cu** | 46.1 ±  2.03  a | 39.72 ±  5.99  a | 48.11 ±  6.94  a | 53.31 ±  5.97  ab | 50.8 ±  4.2  a | 56.25 ±  8.83  ab | 51.66 ± 7.35a | 65.84 ±  13.14  b | 62.39 ± 7.11  a# | 68.54 ±  9  b# |
|  | **Zn** | 0.78 ±  0.21  # | 0.55 ±  0.15 | 0.54 ±  0.04 | 0.59 ±  0.16 | 0.68 ±  0.06 | 0.65 ±  0.24 | 0.62 ± 0.13 | 0.61 ±  0.12 | 0.65 ±  0.12 | 0.73 ±  0.09 |
|  | **Cd** | BMQL | BMQL | BMQL | BMQL | BMQL | BMQL | BMQL | BMQL | BMQL | BMQL |
|  | **Ca** | 104.38 ±  59.2  a# | 47.86 ±  17.07  a | 29.11 ±  1.77  b | 29.2 ±  5.09  a | 49.32 ±  16.46  ab | 44.46 ±  16.74  a | 43.6 ± 12.98b | 34.94 ±  8.27  a | 33.02 ± 8.53  b | 32.77 ±  4.36  a |
|  | **K** | 385.44 ±  21.84 | 362.89 ±  57.76 | 372.85 ±  18.96 | 394.11 ±  18.84 | 357.85 ±  29.09 | 346.99 ±  29.71 | 337.63 ± 32.19 | 336.91 ±  11.95 | 332.56 ± 42.1 | 348.01 ±  28.38 |
|  | **Mg** | 60.89 ±  3.07 | 56.4 ±  8.02 | 57.75 ±  5.32 | 61.14 ±  5.72 | 57.66 ±  7.84 | 57.57 ±  1.97 | 61.07 ±  5.06 | 58.09 ±  2.45 | 58.17 ±  8.83 | 58.01 ±  7.95 |
|  | **Na** | 91.1 ±  7.53 | 72.84 ±  16.77 | 81.95 ±  9.6 | 84.01 ±  7.3 | 85.14 ±  4.52 | 68.2 ±  8.99 | 82.41 ±  7.23 | 81.23 ±  10.46 | 83.26 ± 13.14 | 84.02 ±  13.86 |
| **20 ºC** |  |  |  |  |  |  |  |  |  |  |  |
| **Muscle** | **Cu** | 54.89 ±  18.63  a | 45.9 ±  15.7  a | 50.63 ±  16.97  a | 58.2 ±  6.69  a | 48.95 ±  9.7  a | 53.43 ±  7.27  a | 39.97 ± 10.93a | 54.51 ±  10.91  a | 37.38 ± 18.17  a | 32.62 ±  4.96a |
|  | **Zn** | 0.44 ±  0.12 | 0.46 ±  0.08 | 0.5 ±  0.15 | 0.61 ±  0.16 | 0.53 ±  0.11 | 0.64 ±  0.12 | 0.43 ± 0.1 | 0.61 ±  0.07 | 0.41 ±  0.1 | 0.54 ±  0.03 |
|  | **Cd** | BMQL | BMQL | BMQL | BMQL | BMQL | 0.88 ±  0.11 | BMQL | 1.09 ±  0.62 | BMQL | 0.61 ±  0.23 |
|  | **Ca** | 41.54 ±  20.38 | 33.89 ±  11.59 | 33.12 ±  11.2 | 37.61 ±  10.31 | 40.57 ±  11.73 | 48.75 ± 1  6.8 | 27.98 ± 10.43 | 40.56 ±  19.88 | 33.74 ± 10.48 | 30.53 ±  8.85 |
|  | **K** | 376.72 ±  61.78 | 388.85 ±  81.75 | 367.99 ±  45.99 | 437.45 ±  23.85 | 366.32 ±  36.97 | 399.92 ±  25.72 | 339.15 ± 38.3 | 387.79 ±  6.04 | 343.28 ± 79.87 | 380.23 ±  41.02 |
|  | **Mg** | 60.61 ±  10.37 | 62.59 ±  11.01 | 61.71 ±  10.42 | 71.4 ±  5.06 | 61.16 ±  7.28 | 65.73 ±  1.76 | 57.53 ±  6.59 | 66.21 ±  2.28 | 59.4 ±  15.21 | 63.95 ±  6.06 |
|  | **Na** | 86.58 ±  26.83 | 80.6 ±  20.6 | 75.37 ±  11.83 | 85.27 ±  9.88 | 73.57 ±  10.14 | 73.2 ±  5.79 | 67.57 ±  7.85 | 79.64 ±  5.16 | 65.14 ±  15.9 | 69.33 ±  12.23 |

**SI-Table 12.** Copper, Cd (nmol/g dw), Zn and electrolyte levels (µmol/g dw) in *Cyprinus carpio* carcass, exposed to a metal mixture for 27 days. Mean ± SD, N=5, letters indicate significant differences (p < 0.05). Asterisks (*) indicate differences between control and metal treatment at each sampling day, the hash (#) indicates differences between the same groups (both control and metal treated) at different temperatures and lowercase letters indicate significant differences of the same group among sampling days at each temperature (p < 0.05).

| **10 ºC** |  | **Day 1** | | **Day 7** | | **Day 14** | | **Day 21** | | **Day 27** | |
| --- | --- | --- | --- | --- | --- | --- | --- | --- | --- | --- | --- |
|  |  | **Control** | **Treatment** | **Control** | **Treatment** | **Control** | **Treatment** | **Control** | **Treatment** | **Control** | **Treatment** |
| **Carcass** | **Cu** | 52.82 ±  3.55  a | 47.61 ±  2.29  ab# | 47.43 ±  4.68  ab | 50.43 ±  6.33  a | 44.64 ±  1.87  abc | 44.98 ±  7.33  ab | 38.86 ±  6.43  bc | 37.09 ±  2.77  b | 35.83 ±  2.93  c | 37.39 ±  7.23  b |
|  | **Zn** | 3.43 ± 0.46 | 3.2 ± 0.25 | 3.01 ± 0.26 | 3.09 ± 0.14 | 2.95 ± 0.35 | 3.33 ± 0.51 | 3.13 ± 0.19 | 3.53 ±0.98 | 2.92 ± 0.25 | 3.29 ± 0.5 |
|  | **Cd** | 0.99 ±  0.18  ab | 1.21 ±  0.13  a | 0.91 ±  0.24  a | 1.89 ±  0.55  ab | 0.87 ±  0.26  a | 2.36 ±  0.6  ab | 0.62 ±  0.54  ab | 0.8 ±  0.2  a# | 0.25 ±  0.23  B | 3.26 ±  1.18  b* |
|  | **Ca** | 771.36 ± 109.52  a | 848.4 ±  83.33  a | 690.5 ± 176.77  ab | 770.07 ± 113.45  a | 686.81 ± 119.26  ab | 683.9 ±  183.61  ab | 481.27 ± 234.63  ab | 345.73 ± 41.94  b# | 295.68 ± 127.54  b | 531.62 ± 255.93  a |
|  | **K** | 262.18 ± 16.7  a# | 279.32 ± 12.05  a# | 252.86 ± 13.26  a# | 263.22 ± 12.99  ab# | 252.04 ± 10.19a  # | 245.28 ±  26.58  ab# | 236.36 ± 15.31  a# | 226.57 ±  10.4  b# | 223.72 ± 5.76  a# | 228.05 ±  18.47  b# |
|  | **Mg** | 57.51 ±  4.15  a# | 60.25 ±  2.33  a# | 55.26 ±  2.34  a# | 56.63 ±  1.8  ab# | 54.4 ±  2.5  a# | 55.06 ±  5.02  ab# | 52.66 ± 3.48  a# | 52.44 ±  2.6  ab# | 51.95 ± 2.29  a# | 50.44 ±  1.92  b# |
|  | **Na** | 236.12 ± 25.35  a# | 229.71 ± 22.31  a# | 221.81 ± 15.38  a# | 222.42 ± 19.12a  # | 218.84 ± 12.05  a# | 192.18 ±  25.55  a# | 207.82 ± 16.59  a# | 195.04 ± 10.48  a# | 194.86 ± 7.49  a# | 198.35 ±  23.74  a# |
| **20 ºC** |  |  |  |  |  |  |  |  |  |  |  |
| **Carcass** | **Cu** | 52.48 ±  5.17  a | 60.42 ±  8.01  a | 46.14 ±  2.21  ab | 50.7 ±  3.26  ab | 48.01 ±  2.08  ab | 47.37 ±  5.72  b | 41.16 ± 4.62  ab | 45.32 ±  2.37  b | 37.35 ± 5.04  b | 40.69 ±  4.26  b |
|  | **Zn** | 3.41 ±  0.95 | 3.95 ±  0.58 | 4.01 ±  0.4 | 4.19 ±  0.41 | 3.82 ±  0.5 | 3.75 ±  0.52 | 3.37 ±  0.31 | 3.82 ±  0.3 | 3.2 ±  0.34 | 4.11 ±  0.32 |
|  | **Cd** | 0.62 ±  0.2  a | 1.87 ±  0.63  a* | 0.58 ±  0.04  a | 3.97 ±  0.34  ab* | 0.7 ±  0.05  a | 5.92 ±  1.47  b* | 0.38 ±  0.21  a | 6.98 ±  0.86  b* | 0.44 ±  0.25  a | 4.3 ±  2.56  ab* |
|  | **Ca** | 976.09 ± 273.62  ab | 1195.45 ± 85.59  a | 1052.5 ± 119.05  a | 1021.83 ± 78.29  a | 1035.12 ± 122.38  a | 957.61 ± 205.18  a | 670.26 ± 235.08  ab | 873.44 ± 79.48  a | 584.05 ± 304.77  b | 433.37 ± 130.67  b |
|  | **K** | 323.96 ±  8.1  a | 326.02 ±  20.1  a | 308.65 ±  8.63  ab | 310.67 ±  9.44  ab | 312.27 ±  9.98  a | 310.49 ±  25.11  ab | 289.78 ± 20.12  ab | 298.36 ±  9.93  ab | 273.98 ± 17.13  b | 274.75 ±  23.74  b |
|  | **Mg** | 69.79 ±  3.99  a | 73.92 ±  3.63  a | 69.11 ±  5.44  a | 68.39 ±  2.38  ab | 67.98 ±  3.21  a | 67.12 ± 5.84  ab | 62.99 ± 2.27  a | 63.85 ±  3.27  b | 61.53 ±  3.5  a | 61.42 ±  5.05  b |
|  | **Na** | 310.41 ±  12.58  a | 307.75 ±  29.18  a | 278.31 ±  22.25  a | 276.73 ±  16.06  ab | 281.56 ±  21.02  a | 270 ±  30.01  ab | 258.02 ± 16.58  a | 247.08 ±  8.38  b | 229.04 ± 11.22  a | 235.22 ±  20.79  b |

**SI-Table 13.** Metal and electrolyte levels multiplied by carcass dw and expressed as nmol (Cu and Cd) or µmol (Zn and electrolytes) per total carcass in *Cyprinus carpio*, exposed to a metal mixture for 27 days. Mean ± SD, N=5. Asterisks (*) indicate differences between control and metal treatment at each sampling day, the hash (#) indicates differences between the same groups (both control and metal treated) at different temperatures and lowercase letters indicate significant differences of the same group among sampling days at each temperature (p < 0.05).

| **10 ºC** |  | **Day 1** | | **Day 7** | | **Day 14** | | **Day 21** | | **Day 27** | |
| --- | --- | --- | --- | --- | --- | --- | --- | --- | --- | --- | --- |
|  |  | **Control** | **Treatment** | **Control** | **Treatment** | **Control** | **Treatment** | **Control** | **Treatment** | **Control** | **Treatment** |
| **Tot**  **Carcass**  **(dw)** | **Cu** | 50.13 ± 9.4  a | 39.77 ± 6.03  a | 49.02 ± 4.75  a | 48.01 ± 6.62  ab | 49.59 ± 13.32  a | 48.81 ± 10.92  ab | 54.61 ± 5.87  a | 58.11 ± 8.07  b | 65.32 ± 6.15  a | 58.6 ± 14.36  b |
|  | **Zn** | 3.26 ± 0.71  ab | 2.66 ± 0.35  a | 3.12 ± 0.37  a | 3 ± 0.71  a | 3.27 ± 0.96  ab | 3.71 ± 1.27  ab | 4.51 ± 1.05  ab | 5.6 ± 1.99  b | 5.36 ± 0.88  b | 5.23 ± 1.52  b |
|  | **Cd** | 0.93 ± 0.21  a | 1 ± 0.11  a | 0.92 ± 0.18  a | 1.74 ± 0.25  abc | 0.91 ± 0.2  a | 2.48 ± 0.36  bc*# | 0.75 ± 0.52  ab | 1.24 ± 0.29  ab# | 0.41 ± 0.34  b*# | 4.22 ±1.31  c* |
|  | **Ca** | 724.03 ± 103.68  a | 703.51 ± 79.21  a# | 703.11 ± 151.97  a# | 734.25 ± 127.42  a | 737.68 ± 130.81  a | 711.97 ±  96.24  a | 629.71 ± 191.33  a | 536.15 ±  33.9  a# | 516.11 ± 127.19  a | 745.03 ± 181.08  a |
|  | **K** | 249.45 ± 51.62  a | 233.47 ± 36.44  a | 263.42 ± 37.58  a | 255.72 ±  60.7  a | 277.07 ± 63.36  ab | 267.03 ±  58.95  a | 339.88 ± 72.28  ab | 354.99 ± 46.06  a | 410.71 ± 57.28  b | 373.48 ± 138.44  a |
|  | **Mg** | 54.88 ± 12.78  a | 50.4 ±  8.07  a | 57.84 ±  10.08  a | 55.39 ±  14.95  a | 59.96 ±  14.63  a | 60.23 ±  14.54  a | 76.54 ± 20.39  ab | 82.14 ±  10.49  a | 95.64 ± 15.83  b | 84.27 ±  36.68  a |
|  | **Na** | 223.95 ± 46.84  a | 190.97 ± 27.18  a | 230.5 ±  29.88  a | 216.15 ± 53.13  ab | 240.01 ± 53.31  ab | 208.79 ±  47.44  ab | 298.85 ± 65.23  ab | 305.58 ± 39.76  ab | 357.21 ± 46.38  b | 320.37 ± 109.13  b |
| **20 ºC** |  |  |  |  |  |  |  |  |  |  |  |
| **Tot**  **Carcass**  **(dw)** | **Cu** | 40.78 ± 5.87  a | 50.55 ± 5.36  a | 47.25 ± 3.31  a | 49.26 ± 5.74  a | 44.14 ± 5.37  a | 46.26 ± 1.95  a | 50.1 ± 4.07  a | 47.35 ± 5.26  a | 54.68 ± 0.61  a | 61.26 ± 5.94  a |
|  | **Zn** | 3.03 ±  0.65  a | 3.3 ±  0.33  a | 4.09 ±  0.33  a | 4.05 ±  0.37  a | 3.51 ±  0.57  a | 3.67 ±  0.41  a | 4.18 ±  0.9  a | 3.99 ±  0.5  a | 4.8 ±  1.43  a | 6.22 ±  0.75  b |
|  | **Cd** | 0.54 ±  0.08  a | 1.55 ±  0.46  a* | 0.59 ±  0.04  a | 3.84 ±  0.32  b* | 0.64 ±  0.06  a | 5.73 ±  1.1  b* | 0.44 ±  0.19  a | 7.26 ±  0.74  b* | 0.58 ±  0.16  a | 6.08 ±  2.61  b* |
|  | **Ca** | 858.01 ± 111.43  ab | 1004.2 ± 95.46  a | 1075.42 ± 101.76  a | 992.38 ± 116.43  a | 942.72 ± 58.39  ab | 928.83 ± 121.97  ab | 787.34 ± 150.91  b | 908.63 ± 68.34  a | 778 ± 177.26  b | 635.08 ±  94.57  b |
|  | **K** | 251.76 ± 45.48  a | 273.54 ± 19.53  a | 315.82 ±  9.42  ab | 301.81 ± 30.17  ab | 287.33 ± 36.85  ab | 305.41 ±  31.08  ab | 355.14 ± 44.01  ab | 312.62 ± 41.37  ab | 409.3 ± 111.53  b | 419.67 ±  80.69  b |
|  | **Mg** | 54.51 ±  8.46  a | 62.16 ±  5.87  a | 70.64 ±  4.15  ab | 66.44 ±  6.62  a | 62.52 ±  8.05  ab | 66.32 ±  10.1  a | 77.59 ± 12.14  ab | 66.91 ±  9.35  a | 92 ±  25.09  b | 94.02 ±  19.45  a |
|  | **Na** | 242.95 ± 40.12  a | 257.74 ± 18.79  a | 284.68 ± 21.01  a | 269.62 ± 38.02  a | 257.8 ±  23.3  a | 265.52 ±  32.32  a | 316.5 ± 40.76  a | 258.65 ± 31.77  a | 343.35 ± 97.75  a | 360.17 ±  74.85  a |
